# Distributed forecasting and ant colony optimization for the bike-sharing rebalancing problem with unserved demands

**DOI:** 10.1371/journal.pone.0226204

**Published:** 2019-12-31

**Authors:** Yiwei Fan, Gang Wang, Xiaoling Lu, Gaobin Wang

**Affiliations:** 1 Center for Applied Statistics, Renmin University of China, Beijing, China; 2 School of Statistics, Renmin University of China, Beijing, China; 3 Department of Decision & Information Sciences, Charlton College of Business, University of Massachusetts Dartmouth, MA, United States of America; 4 Invesco Great Wall Fund Management, Shenzhen, China; HEC Montréal, CANADA

## Abstract

Bike-sharing systems (BSS) have widely spread over many cities in the world as an environmentally friendly means to reduce air pollution and traffic congestion. This paper focuses on the bike-sharing rebalancing problem (BRP), which consists of two aspects: determining desired demands at each station and designing routes to redistribute bikes among stations. For the first task, we firstly apply the random forest, a very efficient machine learning algorithm, to forecast desired demands for each station, which can be easily implemented with distributed computing. For the second task, it belongs to the broad class of the vehicle routing problem with pickup and delivery (VRPPD). In most existing settings, all of the demands being strictly satisfied can lead to longer routes and add operational costs. In this paper, we propose a new model with unserved demands by relaxing demands satisfying constraints. Then, we design a distributed ant colony optimization (ACO) based algorithm with some specific modifications to increase its efficiency for the proposed model. We propose to use the percentage of average cost saving per bike as a metric to evaluate the performance of our method on cost-reducing and compare with existing methods and best-known values. Computational results on benchmarks show the advantage of our approach. Finally, we provide a real case study of BSS in Hangzhou, China, with insightful elaborations.

## 1 Introduction

As an emerging green transport, bike-sharing systems (BSS) have expanded in many cities around the world [1]. The BSS offers bikes for public use with high convenience because it allows customers to rent a bike at any self-serve bike station and return it to another. As information technology advances, customers can unlock bikes by scanning QR codes with mobile devices at parking areas without docks instead of placing bikes at fixed stations with docks. Such convenient rent-and-return operations result in a significant increase in the number of bike stations in a city. For example, since New York City launched its Citi Bike program, the number of bike stations has increased from 330 to 750 between August 2015 and January 2019 with more than 12,000 bikes (https://www.citibikenyc.com). As of January 2019, OFO has approximately 10 million bikes in more than 250 cities around 20 countries (http://www.ofo.so), while Mobike has 7 million bikes in more than 180 cities around nine countries (https://mobike.com/). Both OFO and Mobike have more than 200 million customers all over the world. Regardless of the kind of BSS, bikes are moved from one station to another each day. System operators perform redistribution of bikes that is called rebalancing to maintain balanced levels of occupation for each station, which results in ever-increasing operating costs.

This paper focuses on the bike-sharing rebalancing problem (BRP), which consists of two main aspects. Firstly, determine desired demands for each station which is generally a forecasting project. To identify the patterns of demands and make an accurate forecast for each station, in this paper, we utilize the random forest, a very efficient machine learning algorithm with many successful applications in different areas [2, 3]. The second task of BRP is to design routes to rebalance bikes, performed by capacitated vehicles at night (the static case, ignoring user activities) or continuously during the daytime (the dynamic case). In this paper, we consider the static case, which belongs to the broad class of the vehicle routing problem with pickup and delivery (VRPPD) [4]. For the VRPPD, customers have delivery or pickup demands. Vehicles, each having a limited capacity, transport objects (a set of commodities) between origins and destinations. Following the notation introduced in [5], the BRP is essentially a one-commodity (bike) many-to-many VRPPD, any station can serve as an origin or a destination for bikes. In real-life applications, the requirement of strictly satisfying all customer demands may lead to longer routes. For example, if some vehicle carries 20 bikes and a nearby station needs 21 bikes to be delivered, the vehicle must pick up one more bike from other stations to satisfy the need of 21 bikes. Based on this situation, one may consider leaving some of the customer demands unserved in exchange for shorter routes. Finding the balance between these two goals, the length of routes and customer demands fulfillment, is one of the extensions of the BRP. We design a new model, tackling it as a variant of the VRPPD, and develop a distributed ant colony optimization (ACO) based algorithm with some specific modifications for the proposed model. Both the random forest and the ACO can be implemented with distributed computing, making our approach possible to solve the BRP in the significant data context. We then propose to use the percentage of average cost saving per bike as a metric to evaluate the performance of our method on cost-reducing and compare with existing methods and best-known values. Computational results on benchmarks show the advantage of our approach. Finally, we provide a case study, BSS in Hangzhou, where the distance matrix is obtained from the API of Gaode Map (https://lbs.amap.com/api) as the actual distance.

This paper is organized as follows. In Section 2, we review existing methods for the BRP in the literature. In Section 3, we propose the new model for the BRP with unserved demands in mathematical formulations. In Section 4, we first introduce the random forest to forecast customer demands and then develop the ACO based algorithm to solve our model. We also discuss distributed computing strategies for both the random forest and the ACO based algorithm. In Section 5, computational results on benchmarks show the strength of our proposed approach. In Section 6, we provide a real case study of BBS. Section 7 concludes.

## 2 Related work

### 2.1 Demand analysis

Determining customer demands for each station, as a forecasting project, has been discussed in the literature. Berbeglia et al. [5] modeled the time evolution of the dynamics of movements and disentangled the spatial patterns to forecast the number of rentals in the city. Rixey [6] developed a robust regression model for the prediction of station ridership and identified many variables that had statistically significant correlations with station-level bike sharing ridership. Schuijbroek et al. [7] modeled the stochastic demand by viewing the inventory at each station as a non-stationary queuing system with a finite capacity, and derived service level requirements using the transient distribution of the availability of bikes and docks. In this paper, we use the random forest to forecast demands as it is efficient and can find explanatory variables for managerial decision making.

### 2.2 Rebalancing operations

Existing solution approaches to find routes in the BRP are mainly derived from methods for the VRPPD, which can be classified into two categories, exact and heuristic algorithms.

#### 2.2.1 Exact algorithms

Exact algorithms enable the finding of optimal solutions but are time-consuming. The exact methods proposed for the VRPPD mainly focus on the branch-and-cut algorithm [8, 9]. As for the BRP, Dell et al. [4] developed tailor-made branch-and-cut algorithms to solve four mixed-integer linear programming formulations. Erdoğan et al. [10] provided a branch-and-cut algorithm using combinatorial Benders cuts. Branch-and-cut algorithms are also discussed in [11] and [12]. The computational results reported are generally obtained from solving the problems with up to 100 nodes.

#### 2.2.2 Heuristic algorithms

Since the VRPPD is NP-hard [13], approximate solutions are preferred. Therefore, many heuristics arise in the literature, including classical heuristics and metaheuristics. Classical heuristics perform a relatively limited exploration of the search space and typically produce good quality solutions within modest computing time. Constructive approaches and clustering-based methods that assign nodes into clusters and then generate routes are typical ones. More reviews about heuristics for the VRPPD can be found in [14] and [15]. Kloimüllner et al. [16] addressed the BRP by a novel simplified problem definition in conjunction with a cluster-first route-second approach. A similar idea is discussed in [7], where a new cluster-first route-second heuristic is developed. As opposed to classical heuristics, metaheuristics deeply search the solution space, which results in solutions with much higher quality. Neural network algorithm [17], tabu search [18], genetic algorithm [19] and simulated annealing [20] are the most popular ones. Ho and Szeto [21] provided a tabu search for the BRP, where several intensification/diversification mechanisms are applied to the solution obtained from tabu search to improve the solution quality. Dell et al. [22] tackled the BRP with a destroy and repair metaheuristic algorithm, which makes use of a new effective constructive heuristic and several local search procedures. Cruz et al. [23] proposed an iterated local search based heuristic to solve the BRP.

Another important metaheuristic is the ant colony optimization (ACO), inspired by an analogy with real ant colonies foraging for good. The ACO is firstly proposed by [24, 25] to solve the traveling salesman problem and then widely applied in practices [26, 27]. Among the earliest studies, Bull et al. [28] presented the ACO for the vehicle routing problem (VRP) with one central depot and identical vehicles. After that, many researchers [29–32] proposed the improved ACO to solve the VRP and show that the ACO was competitive with other metaheuristics. As for the VRPPD, Gajpal and Abad [33] used the ACO with a construction rule as well as two multi-route local search schemes to solve it. Falcon et al. [34] studied the ACO for the one-commodity traveling salesman problem with selective pickup and delivery within reasonable time and space constraints. Çatay [35] proposed an ACO employing a new saving-based visibility function and a pheromone updating procedure. The numerical tests with the well-known benchmark problems from the literature show that the proposed approach provides competitive results. Different from rich applications in the VRP and the VRPPD, utilizing the ACO to solve the BRP is still limited. Di et al. [36] proposed a novel hybrid approach, combining the constraint programming and the ACO to solve the BRP, while the computational study is not comprehensive. Considering the encouraging results obtained in the VRP and the VRPPD, the application of the ACO to the BRP is an interesting topic.

### 2.3 Unserved demands in the BRP

The BRP with unserved demands is primarily a relaxation formulation of demands satisfying constraints. Relaxation formulations are considered in existing literature, either allowing split deliveries or assuming that vehicles can visit each station an arbitrary number of times. Papazek et al. [37] addressed the problem of finding efficient vehicle tours by minimizing the objective function, consisting of demands deviations, loading activities, and the overall tour lengths. Raviv et al. [38] proposed mixed inter programming models, considering a convex penalty objective function minimizing user dissatisfaction which is linked to the inventory level of each station and tour lengths. Both of them belong to rich static rebalancing problems as introduced in [4], while the setting for our paper is more similar to the VRPPD, where each station is served once by one vehicle. Under this setting, the focus of our paper is to find a balance between the length of routes and demands fulfillment. The problem is tackled as a weighted sum scalarization of multi-objective optimization where the ACO has been widely applied [39, 40]. Besides, the characteristic that the ACO is easily implemented with distributed computing is also one of the motivations that we consider it to solve our proposed problem.

## 3 The BRP with unserved demands

### 3.1 Setting and notation

Consider a graph *G* = (*N*, *A*), where *N* = {*v*_0_} ∪ *V* with *v*_0_ representing the depot, *V* = {*v*_1_, *v*_2_, ⋯, *v*_*n*_} is the set of *n* stations, and *A* = {(*i*, *j*):*i*, *j* ∈ *N*} is the set of edges that connect all stations and the depot. The distance matrix is denoted by *D* = (*d*_*ij*_)_(*n*+1)×(*n*+1)_ with distance *d*_*ij*_(*i*, *j* ∈ *N*) assigned to the edge (*i*, *j*). The demand at the station *i* is denoted by *y*_*i*_, *i* ∈ *V*, where *y*_*i*_ > 0 means |*y*_*i*_| bikes to be picked up and *y*_*i*_ < 0 represents |*y*_*i*_| bikes to be delivered. Assume that there are *K* vehicles in total with the maximum capacity *C*. Define a binary variable *u*_*ij*_, taking value 1 if the edge (*i*, *j*) is traveled by a vehicle, and 0 otherwise. To guarantee that demands are satisfied and vehicle capacities are not exceeded, we introduce an additional variable *f*_*ij*_, which is the number of bikes traveling from *i* to *j*, also called the flow over the edge (*i*, *j*). We summarize the notations as follows.

**Sets**
*N* = set of the depot and stations
*A* = set of edges

**Parameters**
*d*_*ij*_ = distance between *i* and *j*
*y*_*i*_ = demand at the station *i*
*K* = number of vehicles
*C* = maximum capacity of a vehicle

**Decision variables**
*u*_*ij*_ = whether the edge (*i*, *j*) is traveled by a vehicle
*f*_*ij*_ = the flow over the edge (*i*, *j*)

### 3.2 Mathematical formulation

The goal of the BRP is to find the shortest route under explicit constraints. In the standard BRP settings, there is no unserved demand allowed. According to [4], the mathematical formulation can be expressed as follows,

$$\text{min}\sum_{i\in N}\sum_{j\in N}d_{ij}u_{ij}$$ (1)

$$s.t.\sum_{i\in N}u_{ij}=1,\;j\in V$$ (2)

$$\sum_{i\in N}u_{ji}=1,\;j\in V$$ (3)

$$\sum_{j\in V}u_{v_{0}j}\leq K$$ (4)

$$\sum_{j\in V}u_{v_{0}j}=\sum_{j\in V}u_{jv_{0}}$$ (5)

$$\sum_{i\in \tilde{V}}\sum_{j\in \tilde{V}}u_{ij}\leq \left|\tilde{V}\right|-1,\;\tilde{V}\subseteq V,\tilde{V}\neq \emptyset$$ (6)

$$\sum_{i\in N}f_{ji}-\sum_{i\in N}f_{ij}=y_{j},\;j\in V$$ (7)

$$\text{max}\left\{0,y_{i},-y_{j}\right\}u_{ij}\leq f_{ij}\leq \text{min}\left\{C,C+y_{i},C-y_{j}\right\}u_{ij},\;\left(i,j\right)\in A$$ (8)

$$u_{ij}\in \left\{0,1\right\},\;i,j\in N.$$ (9)

The objective function (1) aims to minimize the total length of all routes. The constraints (2) and (3) represent that each station in *V* is served once by only one vehicle. The constraints (4) and (5) ensure, respectively, that at most *K* vehicles leave the depot and, that all vehicles that are used return to the depot at the end of their route. The constraints (6) describe the classical subtour elimination constraints [4], imposing the connectivity of the solution. The constraints (7) ensure the balance of the entering and leaving flow. The constraints (8) guarantee that the demands of each station are satisfied, and vehicle capacities are not exceeded.

Furthermore, we give the following proposition to check the feasibility of a constructed route, which is useful for heuristic algorithms. Similar properties are also discussed in [22] and [41]. The proof is given in S1 Appendix.

**Proposition 1** *For a route o*, *which is an ordered sequence o*_1_, *o*_2_, ⋯, *o*_|*o*|_ *with o*_1_ = *o*_|*o*|_ = *v*_0_ *and o*_*i*_ ∈ *V*, *i* = 2, ⋯, |*o*| − 1,

*(i) it is feasible if and only if* $C-\text{max}\left\{0,{\text{max}}_{2\leq i\leq |o|-1}\sum_{j=2}^{i}y_{o_{j}}\right\}+\text{min}\left\{0,{\text{min}}_{2\leq i\leq |o|-1}\sum_{j=2}^{i}y_{o_{j}}\right\}\geq 0$;

*(ii) the initial number of bikes on a vehicle after it left the depot*, *denoted as z*, *can be any value in the range* $\left[-\text{min}\left\{0,{\text{min}}_{2\leq i\leq |o|-1}\sum_{j=2}^{i}y_{o_{j}}\right\},C-\text{max}\left\{0,{\text{max}}_{2\leq i\leq |o|-1}\sum_{j=2}^{i}y_{o_{j}}\right\}\right]$.

As we stated in Section 1, if customer demands must be met strictly, it is likely that the length of routes will be greatly increased to satisfy a few demands. For example, after serving a certain station, there are 20 bikes carried on the vehicle, and the next closest station needs 21 bikes to be delivered. If the demands are required to be strictly met, the vehicle can only serve the remaining stations first, which may be far away. To avoid this situation, we relax the constraints (7) and (8) by introducing slack variables *s*_*i*_, *i* ∈ *V*. Note that *s*_*i*_ denotes the number of bikes that the station *i* is unserved. Corresponding to the sign of *y*_*i*_, *s*_*i*_ > 0 means that there are *s*_*i*_ bikes unserved for the pickup service, and *s*_*i*_ < 0 means there are |*s*_*i*_| bikes unserved for the delivery service. *s*_*i*_ = 0 means that the demands can be fully satisfied. The smaller |*s*_*i*_|, the more the demands are satisfied. The relaxation formulation of the BRP with unserved demands can be expressed as follows,

$$\text{min}\sum_{i\in N}\sum_{j\in N}d_{ij}u_{ij}+\lambda \sum_{i\in V}\left|s_{i}\right|$$ (10)

$$s.t.\sum_{i\in N}u_{ij}=1,\;j\in V$$ (11)

$$\sum_{i\in N}u_{ji}=1,\;j\in V$$ (12)

$$\sum_{j\in V}u_{v_{0}j}\leq K$$ (13)

$$\sum_{j\in V}u_{v_{0}j}=\sum_{j\in V}u_{jv_{0}}$$ (14)

$$\sum_{i\in \tilde{V}}\sum_{j\in \tilde{V}}u_{ij}\leq \left|\tilde{V}\right|-1,\;\tilde{V}\subseteq V,\tilde{V}\neq \emptyset$$ (15)

$$\sum_{i\in N}f_{ji}-\sum_{i\in N}f_{ij}=y_{j}-s_{j},\;j\in V$$ (16)

$$\text{max}\left\{0,y_{i}-s_{i},-y_{j}+s_{j}\right\}u_{ij}\leq f_{ij}\leq \text{min}\left\{C,C+y_{i}-s_{i},C-y_{j}+s_{j}\right\}u_{ij},\left(i,j\right)\in A$$ (17)

$$u_{ij}\in \left\{0,1\right\},\;i,j\in N.$$ (18)

The objective function (10) consists of two parts, where the first item represents the length of routes and the second item is the number of bikes unserved for all stations. It can be seen as a weighted sum scalarization of multi-objective optimization, where λ is a given parameter to balance the relative importance of the length of routes and customer demands fulfillment. The constraints (11)–(15) are the same as (2)–(6). The constraints (16) and (17) are relaxations of (7) and (8) after introducing slack variables. Note that for a given route, the initial number of bikes on a vehicle, denoted as *z* is adaptively determined by the constructed route, which can be computed according to the following proposition. The proof is given in S2 Appendix.

**Proposition 2** *For a route o*, *which is an ordered sequence o*_1_, *o*_2_, ⋯, *o*_|*o*|_ *with o*_1_ = *o*_|*o*|_ = *v*_0_ *and o*_*i*_ ∈ *V*, *i* = 2, ⋯, |*o*| − 1, *if it is infeasible by Proposition 1 (1)*, *then there are unserved demands along the route*. *Denote* 2 ≤ *i*_1_ ≤ |*o*| − 1 *as the minimum value satisfying* $C-\text{max}\left\{0,{\text{max}}_{2\leq i\leq i_{1}}\sum_{j=2}^{i}y_{o_{j}}\right\}<-\text{min}\left\{0,{\text{min}}_{2\leq i\leq i_{1}}\sum_{j=2}^{i}y_{o_{j}}\right\}$, *then the initial number of bikes on a vehicle z to achieve the minimal unserved demands can be computed as*

$$z=\{\begin{array}{c} \text{max}\{0,C-\text{max}\left\{0,\underset{2\leq i\leq i_{1}}{\text{max}}\sum_{j=2}^{i}y_{o_{j}}\right\}\},\text{if}\;\text{arg}\underset{2\leq i\leq i_{1}}{\text{max}}\sum_{j=2}^{i}y_{o_{j}}<\text{arg}\underset{2\leq i\leq i_{1}}{\text{min}}\sum_{j=2}^{i}y_{o_{j}}\\ \text{min}\{C,-\text{min}\left\{0,{\text{min}}_{2\leq i\leq i_{1}}\sum_{j=2}^{i}y_{o_{j}}\right\}\},otherwise. \end{array}$$

## 4 Solution methodology

In this section, we first introduce the random forest to forecast demands for each station and then propose the ACO based algorithm for solving our proposed model BRP (10)–(18). Moreover, to reduce computational time, we discuss that our proposed algorithms can execute well with distributed computing.

### 4.1 Random forest for forecasting customer demands

Remind that *V* = {*v*_1_, *v*_2_, …, *v*_*n*_} is the set of *n* stations. Denote $Z_{i}={\left\{\left(x_{i}^{\left(t\right)},y_{i}^{\left(t\right)}\right)\right\}}_{t=1}^{T},i\in V$ as the observational dataset for the station *i* with the size *T*, where $x_{i}^{\left(t\right)}={\left(x_{i1}^{\left(t\right)},\cdots ,x_{ip}^{\left(t\right)}\right)}^{T}$ is the *p*-dimensional vector of predictors at time *t* for the station *i* including the historical records and related information, such as weather and dates, and $y_{i}^{\left(t\right)}$ represents demands at time *t* for the station *i*. For each station *i*, we fit a model on the dataset $Z_{i}$ and forecast its demands at time (*T* + 1).

As shown in literature, historical records, weather conditions, and date information have significant effects on customer demands [42]. In the case study of Hangzhou bike-sharing system in Section 6, we calculate the autocorrelation coefficients of the sequence $\left\{y_{i}^{\left(t\right)},t=1,\cdots ,T\right\}$ for *i* ∈ *V* and find the autocorrelation can be traced back to the eighth order, thus the historical demands of the first 8 days are included in the predictors. Moreover, we crawled the weather records such as the temperature, the dew point, the humidity, the air pressure, the wind speed, and whether it rained. We also add the day of the week and whether it is a holiday to the predictors as the date information.

Random forest is an integrated algorithm proposed by [2]. To evaluate the performance of our model, the dataset $Z_{i}$ is divided into a training set to build the model and a test set to evaluate the performance [43]. The random forest first builds *R* uncorrelated decision trees on the training set, each using a part of samples and a part of predictors. Since the response variable for this problem is continuous, we develop a regression tree in two steps below:

Step 1 Divide the predictor space into several distinct and non-overlapping regions.
Step 2 For observations that fall into the same region, we make the same prediction, which is simply the mean of the response values for the training observations in that region.

In Step 1, the regions are likely to have any shape. We choose to divide the predictor space into high-dimensional rectangles and use a top-down, greedy approach that is known as recursive binary splitting. For continuous variables, the first step is to find *j* and $\tilde{x}$ that minimize

$$\sum_{t:x_{ij}^{\left(t\right)}<\tilde{x}}{\left(y_{i}^{\left(t\right)}-{\bar{y}}_{r_{1}}\right)}^{2}+\sum_{t:x_{ij}^{\left(t\right)}\geq \tilde{x}}{\left(y_{i}^{\left(t\right)}-{\bar{y}}_{r_{2}}\right)}^{2},$$ (19)

where ${\bar{y}}_{r_{1}}$ is the mean response for the training observations in $\left\{t:x_{ij}^{\left(t\right)}<\tilde{x}\right\}$, and ${\bar{y}}_{r_{2}}$ is the mean response for the training observations in $\left\{t:x_{ij}^{\left(t\right)}\geq \tilde{x}\right\}$. For category variables, we find the partition of all classes that minimizes the sum of squared errors similar to (19). Next, we split one of the two previously identified regions and repeat the process until a stopping criterion is satisfied. After constructing all tress, the *R* regression trees are then combined to forecast without high variation and instability. The specific steps are shown in Algorithm 1.

**Algorithm 1** Forecasting customer demands by the random forest

1. For *r* = 1, ⋯, *R*,

 (1) In the training set, the Bootstrap method is used to extract *n*_tr_ samples to form a dataset I, where *n*_tr_ is the sample size of the training set.

 (2) Randomly take p* from all *p* predictors. Based on the dataset I and the p* predictors, construct a regression tree *H*_*r*_ using the following two steps.

  a. Find *j* and $\tilde{x}$ that minimize (19), and divide the space into two regions $\left\{t:x_{ij}^{\left(t\right)}<\tilde{x}\right\}$ and $\left\{t:x_{ij}^{\left(t\right)}\geq \tilde{x}\right\}$.

  b. Split $\left\{t:x_{ij}^{\left(t\right)}<\tilde{x}\right\}$ or $\left\{t:x_{ij}^{\left(t\right)}\geq \tilde{x}\right\}$ recursively until the number of samples of each node reaching the minimum value *n*_min_ specified in advance.

2. Output the combined *R* trees.

3. For the new sample with predictor vector $x_{i}^{\left(T+1\right)}$, the prediction result is

$${\hat{y}}_{i}^{\left(T+1\right)}=\frac{1}{R}\sum_{r=1}^{R}H_{r}\left(x_{i}^{\left(T+1\right)}\right),$$

where $H_{r}\left(x_{i}^{\left(T+1\right)}\right)$ is the mean of the response values for the training observations in the region that $x_{i}^{\left(T+1\right)}$ locates at on the *r*-th tree.

Due to the regression nature of demands forecasting, we recommend p* = *p*/3, *n*_min_ = 5 as suggested by [2]. Finally, we choose the root mean square error (RMSE) on the test set as the evaluation measure of the model, that is,

$$RMSE={\left[\frac{1}{n_{\text{te}}}\sum_{\text{test}\;\text{set}}{\left({\hat{y}}_{i}^{\left(t\right)}-y_{i}^{\left(t\right)}\right)}^{2}\right]}^{1/2},$$

where ${\hat{y}}_{i}^{\left(t\right)}$ denotes the predicting value and *n*_te_ represents the sample size of the test set.

### 4.2 Improved ant colony optimization

Based on the ACO stated by [24], we propose the following algorithm to solve the BRP (10)–(18). Firstly, a new heuristic function is defined to incorporate unserved demands. Secondly, several route improvement strategies are considered including parallel route construction, *l*-opt local search, mutation operation, and ant-weight pheromone updating, which are inspired from the ACO for the VRP/VRPPD in literature [28–30, 32, 33] with some modifications to adapt to the new model.

#### 4.2.1 Parallel route construction

During the route construction, we abandon the traditional sequential strategy, where the vehicles leave the depot, construct routes, and return to the depot in order. Instead, we adopt a parallel strategy where all vehicles construct routes at the same time, inspired by [30]. Mazzeo and Loiseau [30] concluded that parallel construction has a better performance than the sequential construction in terms of the objective value.

Assume there are *M* ⋅ *K* ants equally divided into *M* groups where *M* is a given parameter. Each ant represents a vehicle, and the *K* ants in the same group collaborate to complete rebalancing operations. In the beginning, all ants are assigned to the depot. The tabu table *Tabu*_*k*,*m*_ of the *k*-th ant (*k* = 1, ⋯, *K*) in the *m*-th group (*m* = 1, ⋯, *M*) is initialized to an empty set. The pheromone concentration of each path is initialized to a constant *τ*_0_, where *τ*_0_ is a considerably small positive value. In each iteration, the *K* ants in the same group communicate with each other and search their routes at the same time. For each group, when all stations have been served, the rebalancing operation is completed and the objective value of the corresponding rebalancing scheme is computed. Thus, for *M* groups, there are *M* kinds of rebalancing schemes and the scheme with the smallest objective value is chosen as the best-found solution in this iteration.

For the *m*-th group, the current station of the *k*-th ant is denoted as *i*_*k*,*m*_. In the next step, we should determine which route to extend, denoted as *k*_next_ and which station to serve, denoted as *i*_next_. Mazzeo and Loiseau [30] suggested firstly choosing the route that benefits the objective function most and then choosing the next station. In this paper, we consider a two-dimensional multinomial distribution of the pair (*k*, *j*) to allow more deeply search in the solution space. Suppose there are totally Λ pairs of (*k*, *j*). For each pair (*k*, *j*), there is a corresponding integer *κ* ∈ {1, 2, ⋯, Λ}. Given the probability of each pair as shown in (20), let *p*(*κ*) = *p*(*k*, *j*). Then we randomly generate an integer *κ*_next_ ∈ {1, 2, ⋯, Λ} according to the probability distribution *p*(*κ*). Thus, the *κ*_next_-th pair is chosen as (*k*_next_, *i*_next_). For example, assume there are 3 pairs of (*k*, *j*), having the probability 1/2, 1/4, 1/4, respectively. Randomly generate an integer *κ*_next_ ∈ {1, 2, 3} according to the probability distribution where *p*(*κ* = 1) = 1/2, *p*(*κ* = 2) = 1/4, *p*(*κ* = 3) = 1/4. Then we choose the *κ*_next_-th pair as (*k*_next_, *i*_next_). Specifically, the probability of (*k*, *j*) is defined as

$$\begin{array}{c} p\left(k,j\right)=\{\begin{array}{cc} \frac{{\left[\tau \left(i_{k,m},j\right)\right]}^{\alpha }{\left[\phi \left(i_{k,m},j\right)\right]}^{\beta }}{\sum_{\tilde{k}=1}^{K}\sum_{w\in J_{\tilde{k},m}}{\left[\tau \left(i_{\tilde{k},m},w\right)\right]}^{\alpha }{\left[\phi \left(i_{\tilde{k},m},w\right)\right]}^{\beta }}, & 1\leq k\leq K,j\in J_{k,m}\\ 0, & 1\leq k\leq K,j\notin J_{k,m}, \end{array} \end{array}$$ (20)

where $J_{k,m}=\left\{j:j\in V,\left(i_{k,m},j\right)\in A\right\}-\cup_{\tilde{k}=1}^{K}Tabu_{\tilde{k},m}$ represents the set of stations that the *k*-th ant of the *m*-th group is allowed to serve, $Tabu_{\tilde{k},m}$ is the set of stations that the $\tilde{k}$-th ant of the *m*-th group has visited, *τ*(*i*_*k*,*m*_, *j*), *ϕ*(*i*_*k*,*m*_, *j*) represent the pheromone concentration and the heuristic information on the edge (*i*_*k*,*m*_, *j*) respectively, *α*, *β* are parameters indicating the importance of the pheromone concentration and the heuristic information. Note that *τ*(*i*_*k*,*m*_, *j*) is specified in the later updating procedure, and *ϕ*(*i*_*k*,*m*_, *j*) is determined by the heuristic algorithm. Considering the slack variables, let

$$\phi \left(i_{k,m},j\right)=\frac{1}{d_{i_{k,m},j}+\lambda (\sum_{i\in Tabu_{k,m}}|s_{i}\left|+\right|s_{j}\left|\right)},$$

where $\sum_{i\in Tabu_{k,m}}|s_{i}\left|+\right|s_{j}|$ is the minimum value of unserved demands on the route of the *k*-th vehicle in the *m*-th group if the next station is chosen as *j*. It can be calculated according to the initial number of bikes on a vehicle *z* by Proposition 2. For clarity, we summarize the steps of parallel route construction in each iteration as Algorithm 2.

**Algorithm 2** Parallel route construction

In each iteration, we construct routes as follows.

 1. Divide *M* ⋅ *K* ants equally into *M* groups and assign all ants to the depot.

 2. For *m* = 1, 2, ⋯, *M*, *k* = 1, 2, ⋯, *K*, initialize *Tabu*_*k*,*m*_ as an empty set.

 3. For each group *m* = 1, 2, ⋯, *M*, randomly generate a pair (*k*_next_, *i*_next_) with probability *p*(*k*, *j*) defined in (20). Then choose the ant *k*_next_ to move to the station *i*_next_. Insert *i*_next_ to $Tabu_{k_{\text{next}},m}$.

 4. Repeat Step3 until all stations have been served for each group, that is, $\cup_{k=1}^{K}Tabu_{k,m}=V$ for *m* = 1, 2, ⋯, *M*.

 5. For *m* = 1, 2, ⋯, *M*, move all ants from the last served station to the depot and compute the objective value of the corresponding scheme, denoted as *G*^(*m*)^. The scheme with the minimum *G*^(*m*)^ is the best found solution in this iteration.

**Fig 1:**
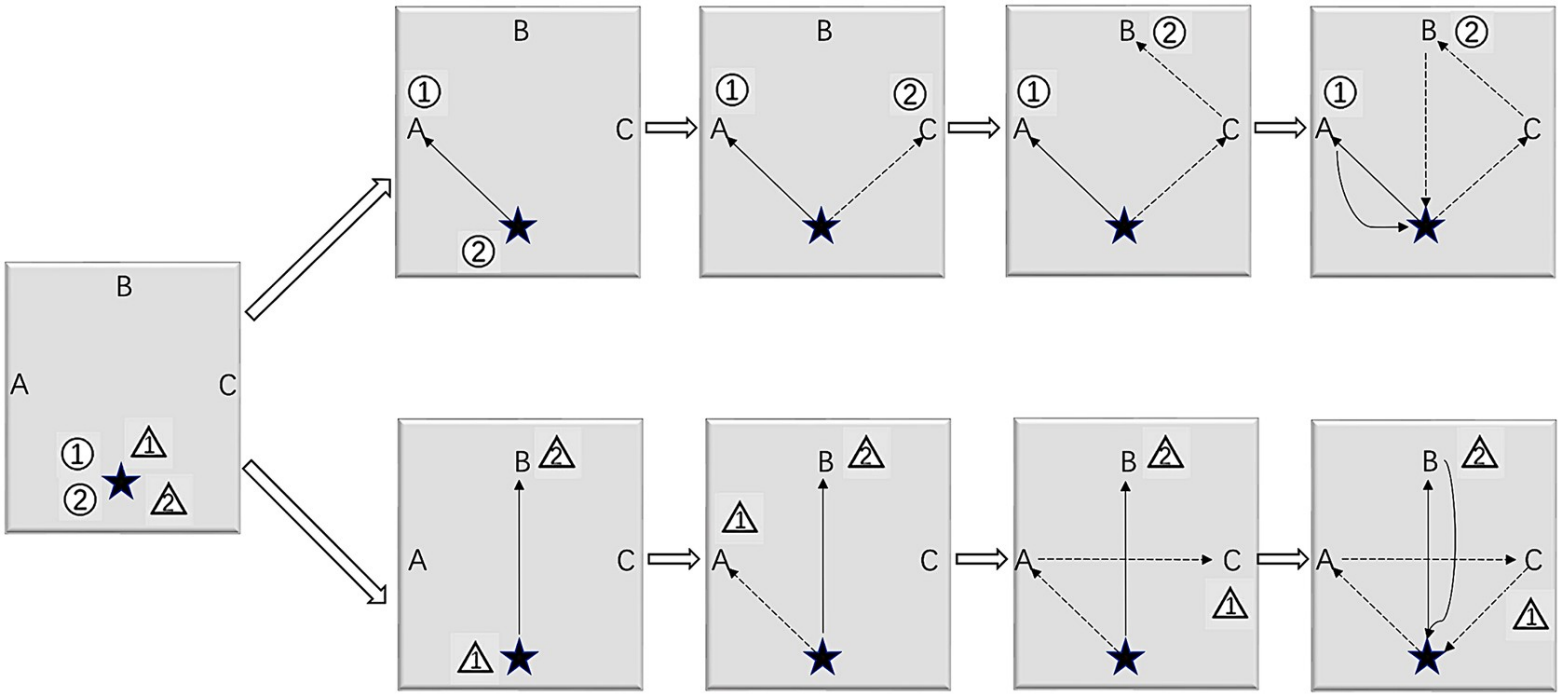
An illustrative example of parallel route construction.

Fig 1 shows an illustrative example. There are three stations labeled as “A”, “B” and “C”. The depot is represented by a star. Suppose *M* = 2, *K* = 2, where the first group is represented by a circle and the second group is represented by a triangle with the digit denoting the ant. In each iteration, all ants are assigned to the depot at the beginning. The construction procedure for two groups are independent which are shown in the first and the second rows, respectively. For the first group, we calculate the probability *p*(1, “A”), *p*(1, “B”), *p*(1, “C”), *p*(2, “A”), *p*(2, “B”), *p*(2, “C”) and generate a pair (*k*_next_, *i*_next_) randomly by these probabilities. Assuming (1, “A”) is generated, move the ① to the station “A”. Then we calculate *p*(1, “B”), *p*(1, “C”), *p*(2, “B”), *p*(2, “C”) and generate a pair (*k*_next_, *i*_next_) randomly. Assuming (2, “C”) is generated, we move the ② to the station “C”. Then we calculate (1, “B”),(2, “B”) and generate a pair (*k*_next_, *i*_next_) randomly. Assuming (2, “B”) is generated, we move the ② to the station “B”. So far, all stations have been served and the rebalancing operation has been completed for the first group. Move all ants to the depot and calculate the objective value *G*^(1)^. A similar procedure is applied to the second group and the objective value obtained is denoted as *G*^(2)^. The rebalancing scheme with a smaller objective value is chosen as the best found solution in this iteration.

#### 4.2.2 Route improvement strategies

After all the groups finish the route construction, several route improvement strategies are applied to the local best-found routes. The first strategy is the *l*-opt heuristic [28, 29, 32]. It is a local search procedure. In each step, *l* links of a current route are replaced by other *l* links, to test if an overall improvement in the objective function can be obtained. We consider 2-opt and 3-opt in this paper.

Another strategy is the mutation operation, an approach to improve the performance significantly, which is first applied to the ACO in [32]. The idea is to mutate the routes, producing a new solution that is not very far from the original one. We consider three mutation operators, insertion mutation, swap mutation, and cross mutation. For insertion mutation, we first choose a station and delete the station in its corresponding route. Then we add it to the end of another chosen route as the last served station before returning to the depot. Swap(*n*_1_,*n*_2_) mutation is to exchange *n*_1_ stations in one route with *n*_2_ stations in another route. We consider Swap(1,1), Swap(2,2), Swap(1,2) and Swap(2,3). Cross mutation is to choose two routes, cut each route into two parts and merge them crossly. After mutation operation, to ensure local optimality, the 2-opt heuristic proceeds for each route.

#### 4.2.3 Ant-weight pheromone updating

Adopting the ant-weight strategy in [32], for ∀*i*, *i*′ ∈ *N*, the updating equation of the pheromone concentration is,

$$\begin{array}{c} \begin{array}{c} \tau^{\text{new}}\left(i,i^{\prime }\right)=q\cdot \tau^{\text{old}}\left(i,i^{\prime }\right)+\sum_{k=1}^{K}\Delta \tau^{k}\left(i,i^{\prime }\right)\\ \Delta \tau^{k}\left(i,i^{\prime }\right)=\{\begin{array}{cc} \frac{Q}{K\cdot G_{op}}\cdot \frac{G_{op}^{k}-d_{ii^{\prime }}}{|op^{k}|\cdot G_{op}^{k}}, & \left(i,i^{\prime }\right)\in op^{k}\\ 0, & \text{otherwise}, \end{array} \end{array} \end{array}$$ (21)

where *q* represents the volatilization rate of the pheromone concentration, *G*_*op*_ represents the best found value of the objective function obtained so far, $G_{op}^{k}$ represents the part that the *k*-th vehicle contributes to *G*_*op*_, *op*^*k*^ is the best found routes of the *k*-th vehicle, |⋅| represents the cardinality and *Q* is a constant. The ant-weight strategy updates the pheromone considering both global information *Q*/(*K* ⋅ *G*_*op*_) and local information $\left(G_{op}^{k}-d_{ii^{\prime }}\right)/(|op^{k}\left|\cdot G_{op}^{k}\right)$, ensuring that the increment of pheromone is directly proportional to the quality of routes. Moreover, to prevent from local optima, we adopt the MAX-MIN strategy [44] where *τ*(*i*, *i*′), *i*, *i*′ ∈ *N* is bounded in [*τ*_min_, *τ*_max_] with

$$\tau_{\text{min}}=\frac{Q}{2\sum_{i\in V}d_{v_{0},i}},\tau_{\text{max}}=\frac{Q}{\sum_{i\in V}d_{v_{0},i}}.$$

The specific steps of the ACO are shown in Algorithm 3.

**Algorithm 3** Designing best found routes by the improved ACO

1. *Initialization*. Set *τ*_0_ as a considerably small positive value.

2. *Parallel route construction*. The *M* ⋅ *K* ants construct routes by Algorithm 2. The rebalancing scheme with the minimum objective value is the local best found solution.

3. *Route improvement*. Several route improvement strategies including 2-opt, 3-opt and mutation operations are applied to the local best found solution.

4. *Pheromone updating*. The pheromone concentration between any two nodes is updated by (21).

5. *Iteration*. Repeat Steps 2-4 until the termination condition is met, which is set to a pre-defined time limitation in this paper.

In terms of computational complexity, the initialization requires *O*(*n*^2^), the parallel route construction requires *O*(*n*^2^ ⋅ *M*), and the pheromone updating requires *O*(*n*^2^), which are as same as the standard ACO proposed in [24]. For route improvement strategies, as discussed in [22], we have *O*(*n*^2^) for 2-opt, *O*(*n*^3^) for 3-opt, *O*(*n*) for the insertion mutation, *O*(*n*^2^) for each swap mutation and *O*(*n*^2^) for the cross mutation.

### 4.3 Distributed computing

In the significant data era, an algorithm is often characterized as being implemented in a distributed computing way. When forecasting customer demands, the random forest model we consider supports distributed computing. In Algorithm 1, the *R* regression trees can be constructed independently in different processors. In each processor, we first extract samples with replacement, called the Bootstrap resampling method, to form a dataset and then randomly take a part of predictors. Based on the extracting samples and predictors, a regression tree is formed in its processor. After the creation of all trees is finished, for a new sample, the forecast is first calculated using a single tree in each processor and then collected to take the average. The machine learning frameworks Mahout of the Hadoop platform and Mllib of the Spark platform have ready-made interfaces of the random forest algorithm. In this paper, we shall use Mahout for distributed computing of the random forest model.

The distributed strategies of the ACO have been discussed in the literature [45–47], among which the most popular ones are the independent strategy, master-slave paradigm, fine-grained, and coarse-grained. The independent strategy executes parallel independent runs of the ACO [48]. In the master-slave paradigm, each ant in the slave finds a solution and sends it to the master. After all the slaves find a solution, the master updates the pheromone information and then sends them back to all slaves [49]. In the fine-grained model, the population of ants is assigned to a large number of tiny groups, maintained by different processors. The information is exchanged frequently among those processors after each iteration [50]. The coarse-grained model is different. The population of ants is divided into a few groups. Different processors exchange information after several fixed numbers of iterations [51]. For the ACO in Section 4.2, we use the fine-grained strategy to implement distributed computing as the route constructions of the *M* groups of ants do not interfere with each other in each iteration. The *M* groups of ants find solutions on different processors simultaneously. After all the groups of ants have finished the creation of routes, the results are collected, and the information among processors is exchanged. Then we continue to update the pheromone information. This distributed strategy makes the ACO computationally efficient.

## 5 Computational study

In this section, we evaluate our method on benchmarks in [4]. There are 65 instances in total collected from the web sites of 22 BSS. The number of stations varies from 13 to 116. Customer demands for each station are given in advance so that the forecasting procedure does not proceed for these instances, and we directly solve the best-found routes. The distance matrix between each pair of points is asymmetric, considering the one-way principle. More details about these benchmarks can be found in [4].

### 5.1 Computational results

Our algorithm was coded in R and run on an Intel Core i7-7500 CPU, 2.70 GHz, 8.00GB. By the number of stations, we divide the instances into three groups, small-size instances for the first 35 instances, medium-size instances for the next 9 instances and large-size instances for the last 21 instances. The stopping criteria are set to 180, 1800, and 3600 CPU seconds for small-size, medium-size and large-size instances, respectively.

For the ACO stated in Section 4.2, some parameters need to be determined. These parameters can be grouped into two categories; one is related to the algorithm itself, the other is linked to the problem studied, both having impacts on the computational results. According to [24, 32] and some preliminary experiments, the first category of parameters is set as *M* = *n*, *α* = 1, *β* = 5, *q* = 0.8, *Q* = 100, *τ*_0_ = 2*Q*/(3∑_*i*∈*V*_ *d*_*v*_0_,*i*_) to achieve a satisfying convergence rate. The second category of parameters includes the capacity of vehicles, that is, *C* as shown in Tables 1–3, and the number of available vehicles which is set as *K* = ⌊∑_*i*∈*V*_ *y*_*i*_/*C*⌋ + 1. Another key parameter is λ, the relative importance of the length of routes and customer demands, which is set in terms of the problem addressed. In Section 5.2, we discuss the effect of different values of λ and suggest to set λ as the 5% quantile of the distance {*d*_*v*_0_, *i*_, *i* ∈ *V*} for the small-size and medium-size instances and 0.5% quantile for the large-size instances, respectively.

**Table 1:** Computational results for small-size instances.

	City (|*N*|, *C*)	∑|*y*_*i*_|	LB	UB	B&C	DR	ACO					
							%*s*	*Gap*_*L*_	*Gap*_*U*_	*Save*_*U*_	*Save*_*B*_	*Save*_*D*_
1	Bari(13,30)	32	14600	14600	14600	14600	0.00	0.00	0.00	0.00	0.00	0.00
2	Bari(13,20)	32	15700	15700	15700	15700	0.00	0.00	0.00	0.00	0.00	0.00
3	Bari(13,10)	32	20600	20600	20600	20600	3.13	-8.74	-8.74	-5.79	-5.79	-5.79
4	ReggioEmilia(14,30)	48	16900	16900	16900	16900	0.00	0.00	0.00	0.00	0.00	0.00
5	ReggioEmilia(14,20)	48	23200	23200	23200	23200	0.00	0.00	0.00	0.00	0.00	0.00
6	ReggioEmilia(14,10)	48	32500	32500	32500	32500	0.00	0.00	0.00	0.00	0.00	0.00
7	Bergamo(15,30)	65	12600	12600	12600	12600	0.00	0.00	0.00	0.00	0.00	0.00
8	Bergamo(15,20)	65	12700	12700	12700	12700	0.00	0.00	0.00	0.00	0.00	0.00
9	Bergamo(15,12)	65	13500	13500	13500	13500	4.62	-4.44	-4.44	0.18	0.18	0.18
10	Parma(15,30)	36	29000	29000	29000	29000	0.00	0.00	0.00	0.00	0.00	0.00
11	Parma(15,20)	36	29000	29000	29000	29000	0.00	0.00	0.00	0.00	0.00	0.00
12	Parma(15,10)	36	32500	32500	32500	32500	2.78	-4.92	-4.92	-2.21	-2.21	-2.21
13	Treviso(18,30)	37	29259	29259	29259	29259	0.00	0.00	0.00	0.00	0.00	0.00
14	Treviso(18,20)	37	29259	29259	29259	29259	0.00	0.00	0.00	0.00	0.00	0.00
15	Treviso(18,10)	37	31443	31443	31443	31443	5.41	-4.63	-4.63	0.82	0.82	0.82
16	LaSpezia(20,30)	49	20746	20746	20746	20746	0.00	0.00	0.00	0.00	0.00	0.00
17	LaSpezia(20,20)	49	20746	20746	20746	20746	0.00	0.00	0.00	0.00	0.00	0.00
18	LaSpezia(20,10)	49	22811	22811	22811	22811	6.12	-8.88	-8.88	-2.94	-2.94	-2.94
19	BuenosAires(21,30)	325	76999	76999	76999	76999	0.12	-0.77	-0.77	-0.65	-0.65	-0.65
20	BuenosAires(21,20)	325	91619	91619	91619	91619	0.62	-6.54	-6.54	-5.96	-5.96	-5.96
21	Ottawa(21,30)	45	16202	16202	16202	16202	0.00	0.00	0.00	0.00	0.00	0.00
22	Ottawa(21,20)	45	16202	16202	16202	16202	0.00	0.00	0.00	0.00	0.00	0.00
23	Ottawa(21,10)	45	17576	17576	17576	17576	0.00	0.06	0.06	0.06	0.06	0.06
24	SanAntonio(23,30)	74	22982	22982	22982	22982	0.00	0.00	0.00	0.00	0.00	0.00
25	SanAntonio(23,20)	74	24007	24007	24007	24007	0.00	1.57	1.57	1.57	1.57	1.57
26	SanAntonio(23,10)	74	40149	40149	40149	40149	1.35	-2.14	-2.14	-0.80	-0.80	-0.80
27	Brescia(27,30)	88	30300	30300	30300	30300	0.00	0.00	0.00	0.00	0.00	0.00
28	Brescia(27,20)	88	31100	31100	31100	31100	0.00	0.00	0.00	0.00	0.00	0.00
29	Brescia(27,11)	88	35200	35200	35200	35200	0.00	0.00	0.00	0.00	0.00	0.00
30	Roma(28,30)	230	61900	61900	61900	61900	0.00	0.32	0.32	0.32	0.32	0.32
31	Roma(28,20)	230	66600	66600	66600	66670	1.30	-2.85	-2.85	-1.57	-1.57	-1.67
32	Roma(28,18)	230	68300	68300	68300	68300	0.43	-0.44	-0.44	0.00	0.00	0.00
33	Madison(28,30)	64	29246	29246	29246	29246	0.00	0.00	0.00	0.00	0.00	0.00
34	Madison(28,20)	64	29839	29839	29839	29839	0.00	0.00	0.00	0.00	0.00	0.00
35	Madison(28,10)	64	33848	33848	33848	33848	0.63	-1.15	-1.15	-0.53	-0.53	-0.53
	*Avg*_*s*_						0.76	-1.24	-1.24	-0.50	-0.50	-0.50

**Table 2:** Computational results for medium-size instances.

	City (|*N*|, *C*)	∑|*y*_*i*_|	LB	UB	B&C	DR	ACO					
							%*s*	*Gap*_*L*_	*Gap*_*U*_	*Save*_*U*_	*Save*_*B*_	*Save*_*D*_
36	Guadalajara(41,30)	62	57476	57476	57476	57476	0.00	0.00	0.00	0.00	0.00	0.00
37	Guadalajara(41,20)	62	59493	59493	59493	59493	0.00	0.00	0.00	0.00	0.00	0.00
38	Guadalajara(41,11)	62	64981	64981	64981	64981	3.23	-2.71	-2.71	0.54	0.54	0.54
39	Dublin(45,30)	148	33548	33548	33548	33595.4	0.00	0.00	0.00	0.00	0.00	-0.14
40	Dublin(45,20)	148	39786	39786	39786	39817.2	0.00	0.03	0.03	0.03	0.03	-0.05
41	Dublin(45,11)	148	54392	54392	54392	55000.6	0.00	0.00	0.00	0.00	0.00	-1.11
42	Denver(51,30)	139	51583	51583	51583	51583	0.00	1.40	1.40	1.40	1.40	1.40
43	Denver(51,20)	139	53465	53465	53465	53465	0.00	0.18	0.18	0.18	0.18	0.18
44	Denver(51,10)	139	67459	67459	67459	67459	1.44	-10.73	-10.73	-9.43	-9.43	-9.43
	*Avg*_*m*_						0.52	-1.31	-1.31	-0.81	-0.81	-0.96

**Table 3:** Computational results for large-size instances.

	City (|*N*|, *C*)	∑|*y*_*i*_|	LB	UB	B&C	DR	ACO					
							%*s*	*Gap*_*L*_	*Gap*_*U*_	*Save*_*U*_	*Save*_*B*_	*Save*_*D*_
45	Rio de J.(55,30)	197	122547	122547	122547	122582.1	1.02	-1.13	-1.13	-0.12	-0.12	-0.15
46	Rio de J.(55,20)	197	155446	155517	156140	155992.7	6.09	-9.65	-9.69	-3.83	-4.22	-4.12
47	Rio de J.(55,10)	197	253690	257147	259049	257412.5	38.07	-62.06	-62.57	-39.57	-40.01	-39.63
48	Boston(59,30)	256	65669	65669	65669	65669	0.00	3.81	3.81	3.81	3.81	3.81
49	Boston(59,20)	256	71879	71879	71879	72057.2	0.00	4.74	4.74	4.74	4.74	4.49
50	Boston(59,16)	256	74790	75065	75065	75318.8	6.25	3.13	2.76	9.61	9.61	9.24
51	Torino(75,30)	231	47634	47634	47634	47634	0.00	2.56	2.56	2.56	2.56	2.56
52	Torino(75,20)	231	50204	50204	50204	51026	0.00	4.75	4.75	4.75	4.75	3.06
53	Torino(75,10)	231	58814	61717	64797	62031.6	8.23	1.98	-2.82	5.89	0.86	5.35
54	Toronto(80,30)	348	40794	41390	41549	41783.5	0.00	9.39	7.81	7.81	7.40	6.80
55	Toronto(80,20)	348	42621	46631	47898	46876.6	0.00	18.65	8.45	8.45	5.58	7.88
56	Toronto(80,12)	348	54238	58539	60763	58878.7	8.33	7.11	-0.76	8.26	4.30	7.64
57	Miami(82,30)	318	152229	154038	156104	154344.7	11.64	-10.38	-11.44	0.22	-1.10	0.03
58	Miami(82,20)	318	209379	214250	229237	215167.1	33.96	-53.85	-54.90	-31.70	-36.17	-31.99
59	Miami(82,10)	318	390536	397921	415762	402746.8	49.37	-78.75	-79.15	-58.82	-60.58	-59.31
60	C. de Mex.(90,30)	611	67894	72279	88227	72797.5	0.98	3.14	-3.11	-2.15	-19.84	-2.85
61	C. de Mex.(90,20)	611	88952	94319	116418	94612.6	5.40	2.15	-3.67	1.83	-17.50	1.52
62	C. de Mex.(90,17)	611	99714	103658	109573	104213.7	9.17	-14.74	-17.98	-9.70	-14.58	-10.19
63	Minneapolis(116,30)	286	136148	137843	137843	139874.3	3.85	-5.96	-7.12	-3.40	-3.40	-4.80
64	Minneapolis(116,20)	286	157736	166150	186449	166797	12.24	-4.59	-9.42	3.21	-8.03	2.81
65	Minneapolis(116,10)	286	246133	262936	298886	264335.2	21.68	-30.82	-35.24	-17.32	-27.26	-17.76
	*Avg*_*l*_						10.30	-10.03	-12.58	-5.02	-9.01	-5.51

We compare our proposed method with the B&C method (the exact method for the BRP without unserved demands proposed in [4] using the branch-and-cut algorithm) and the DR method (the heuristic method for the BRP without unserved demands proposed in [22] using the destroy and repair algorithm) and best-known values (the best result for each instance among the above two existing methods). We propose to use the percentage of average cost saving per bike as a metric to evaluate the performance of our method on cost-reducing, where the definition will be given soon.

The average computational results over 5 runs are shown in Tables 1, 2 and 3. The following information is provided for each instance:

- City (|*N*|, *C*): The name of the city together with the number of stations and the capacity of each vehicle.
- ∑|*y*_*i*_|: The total demands for all stations.
- LB: The lower bound of the best-found length of routes (in meters) without unserved demands provided by the branch-and-cut algorithm [4]. Note that the values could be of infeasible solutions.
- UB: The best-known length of routes (in meters) without unserved demands, obtained from the two existing methods, the BRP [4] and the DR [22].
- B&C: The best-found length of routes (in meters) without unserved demands for the B&C method [4].
- DR: The best-found length of routes (in meters) without unserved demands for the DR method [22].
- ACO %*s*: The percentage of unserved demands, calculated as 100 × ∑|*s*_*i*_|/∑|*y*_*i*_|.
- ACO *Gap*_*L*_: The percentage of route length saving by our method compared with the lower bound, calculated as 100 × (*L*_*bf*_ − *LB*)/*LB* where *L*_*bf*_ is the best-found route length of our proposed method, which is not shown for save of space.
- ACO *Gap*_*U*_: The percentage of route length saving by our method compared with the best-known results, which can be calculated as 100 × (*L*_*bf*_ − *UB*)/*UB*. The smaller, the better.
- ACO *Save*_*U*_: The percentage of average cost saving per bike by our method compared with the best-known values, which can be calculated as *Save*_*U*_ = 100 × (*Avecost* − *Avecost*_0_)/*Avecost*_0_, where *Avecost*_0_ = *UB*/∑|*y*_*i*_| is the average cost of each bike for the best-known values and *Avecost* = *L*_*bf*_/(∑|*y*_*i*_| − ∑|*s*_*i*_|) is the average cost of our method. The smaller, the better.
- ACO *Save*_*B*_: The percentage of average cost saving per bike by our method compared with the B&C method, calculated similarly as *Save*_*U*_ with *Avecost*_0_ being computed with best-found results of the B&C method rather than UB.
- ACO *Save*_*D*_: The percentage of average cost saving per bike by our method compared with the DR method, calculated similarly as *Save*_*U*_ with *Avecost*_0_ being computed with the best-found results of the DR method rather than UB.

As shown in Tables 1, 2 and 3, when unserved demands are allowed, for some instances, the length of best-found routes can be greatly reduced by sacrificing a small number of customer demands. Take the 3-rd instance Bari(13,10) as an example. The length of routes has been reduced by about 8.74% compared with the best-known value, leaving only 3.13% unserved. Also, the percentage of cost-saving achieves 5.79%. For instances that there are no unserved demands involved (%*s* = 0.00), the ACO can achieve results comparable to UB by presenting a small value of *Gap*_*U*_. *Avg*_*s*_, *Avg*_*m*_ and *Avg*_*l*_ denote the average results of the small-size, medium-size and large-size instances, respectively. The average percentage of unserved demands for the small-size instances is only 0.76%, and the length of best-found routes is improved by 1.24%. The percentage of cost-saving is 0.50%. For medium-size instances, the average percentage of unserved demands is 0.52%, and the length of best-found routes is improved by 1.31% with the percentage of cost-saving being 0.81%, 0.81% and 0.96% for UB, B&C, and DR, respectively. For large-size instances, our method leads to more unserved demands and much shorter routes, especially when the capacity of vehicles is small. The average percentage of unserved demands is 10.30%, and the average percentage of reduction of route lengths achieves 12.58%. The percentage of cost-saving is 5.02%, 9.01% and 5.51% compared with UB, B&C, and DR, respectively.

**Fig 2:**
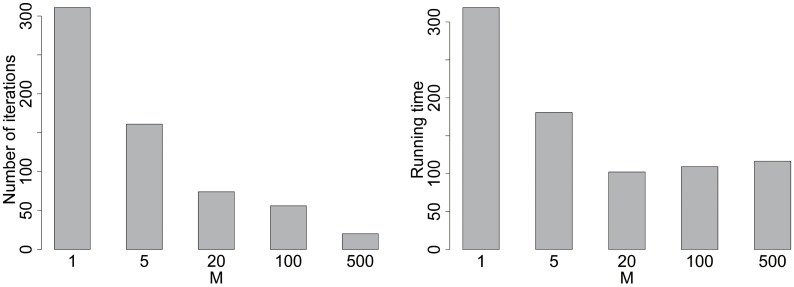
Effects of *M* for the instance LaSpezia(20,30). (a) The number of iterations that the ACO first achieves the best found solution; (b) The running time that the ACO first achieves the best found solution.

Next, we discuss the effect of *M* on the proposed algorithm. Note that in each iteration, there are *M* groups of ants search routes and the best-found route among the *M* groups is chosen as the local solution in this iteration. When *M* increases, it is more probable to find better routes in each iteration while the running time for each iteration is also increasing. On the one hand, we prefer a deep search in each iteration. On another hand, the pheromone updating after each iteration contributes to the convergence of the ACO, and we prefer less computational time for each iteration. Thus, the choice of *M* is the trade-off of these two aspects. In the computational studies, we set *M* = *n* according to existing literature and some preliminary experiments. To explore the effect of *M* on the computational results, Fig 2 shows the number of iterations and the running time that the ACO first achieves the best-found solution of the sixteenth instance in the benchmark as an example. One can see when *M* increases, the number of iterations is decreasing significantly. Moreover, the running time is decreasing first and then increased slightly. It is reasonable since, for small *M*, it requires a large number of iterations to achieve the best-found solution, while for large *M*, the time cost for each iteration is considerably high. Thus, we recommend a moderate value of *M* to balance the number of iterations and the time cost for each iteration.

### 5.2 Effect of unserved demands

In this subsection, we set λ as different values to discuss its impact on results. Other parameters are set as same as those in Section 5.1. For λ, we consider λ = 0.5%, 1%, 2%, 5% and 10% quantile of $\left\{d_{v_{0},i},i\in V\right\}$ for all instances. The average computational results over 5 runs are shown in Tables 4, 5 and 6. The definitions of %*s*, *Gap*_*U*_, and *Save*_*U*_ are as same as those in Section 5.1, representing the percentage of unserved demands, the percentage of route length saving by our method compared with the best-known values, and the percentage of average cost saving per bike by our method compared with the best-known values, respectively.

**Table 4:** Computational results for small-size instances with different values of λ.

	0.5% quantile			1% quantile			2% quantile			5% quantile			10% quantile		
	%*s*	*Gap*_*U*_	*Save*_*U*_	%*s*	*Gap*_*U*_	*Save*_*U*_	%*s*	*Gap*_*U*_	*Save*_*U*_	%*s*	*Gap*_*U*_	*Save*_*U*_	%*s*	*Gap*_*U*_	*Save*_*U*_
1	0.00	0.00	0.00	0.00	0.00	0.00	0.00	0.00	0.00	0.00	0.00	0.00	0.00	0.00	0.00
2	15.63	-7.01	10.21	15.63	-7.01	10.21	15.63	-7.01	10.21	0.00	0.00	0.00	0.00	0.00	0.00
3	46.88	-29.13	33.41	46.88	-29.13	33.41	46.88	-29.13	33.41	3.13	-8.74	-5.79	3.13	-8.74	-5.79
4	0.00	0.00	0.00	0.00	0.00	0.00	0.00	0.00	0.00	0.00	0.00	0.00	0.00	0.00	0.00
5	18.75	-27.16	-10.34	18.75	-27.16	-10.34	18.75	-27.16	-10.34	0.00	0.00	0.00	0.00	0.00	0.00
6	39.58	-48.00	-13.93	39.58	-48.00	-13.93	39.58	-48.00	-13.93	0.00	0.00	0.00	0.00	0.00	0.00
7	0.00	0.00	0.00	0.00	0.00	0.00	0.00	0.00	0.00	0.00	0.00	0.00	0.00	0.00	0.00
8	6.15	-0.79	5.72	0.00	0.00	0.00	0.00	0.00	0.00	0.00	0.00	0.00	0.00	0.00	0.00
9	20.00	-6.67	16.67	12.31	-5.93	7.28	4.62	-4.44	0.18	4.62	-4.44	0.18	0.00	0.00	0.00
10	0.00	0.00	0.00	0.00	0.00	0.00	0.00	0.00	0.00	0.00	0.00	0.00	0.00	0.00	0.00
11	0.00	0.00	0.00	0.00	0.00	0.00	0.00	0.00	0.00	0.00	0.00	0.00	0.00	0.00	0.00
12	16.67	-9.23	8.92	16.67	-10.77	7.08	16.67	-10.77	7.08	2.78	-4.92	-2.21	2.78	-4.92	-2.21
13	0.00	0.00	0.00	0.00	0.00	0.00	0.00	0.00	0.00	0.00	0.00	0.00	0.00	0.00	0.00
14	0.00	0.00	0.00	0.00	0.00	0.00	0.00	0.00	0.00	0.00	0.00	0.00	0.00	0.00	0.00
15	13.51	-6.94	7.60	13.51	-6.94	7.60	5.41	-2.62	2.94	5.41	-4.63	0.82	0.00	0.00	0.00
16	0.00	0.00	0.00	0.00	0.00	0.00	0.00	0.00	0.00	0.00	0.00	0.00	0.00	0.00	0.00
17	0.00	0.00	0.00	0.00	0.00	0.00	0.00	0.00	0.00	0.00	0.00	0.00	0.00	0.00	0.00
18	6.12	-8.88	-2.94	6.12	-8.88	-2.94	6.12	-8.88	-2.94	6.12	-8.88	-2.94	0.00	0.00	0.00
19	18.46	-29.94	-14.08	5.17	-15.15	-10.52	3.08	-11.53	-8.72	0.12	-0.77	-0.65	0.00	0.00	0.00
20	16.62	-31.84	-18.26	8.74	-24.29	-17.04	2.46	-13.71	-11.53	0.62	-6.54	-5.96	0.62	-6.54	-5.96
21	0.00	0.00	0.00	0.00	0.00	0.00	0.00	0.00	0.00	0.00	0.00	0.00	0.00	0.00	0.00
22	0.00	0.00	0.00	0.00	0.00	0.00	0.00	0.00	0.00	0.00	0.00	0.00	0.00	0.00	0.00
23	4.44	-7.50	-3.20	4.44	-7.03	-2.70	4.44	-6.71	-2.37	0.00	0.06	0.06	0.00	0.00	0.00
24	13.51	-21.37	-9.08	13.51	-21.92	-9.72	0.00	0.00	0.00	0.00	0.00	0.00	0.00	0.00	0.00
25	27.03	-26.06	1.33	27.03	-24.85	2.98	1.35	0.86	2.24	0.00	1.57	1.57	0.00	1.57	1.57
26	40.54	-54.99	-24.30	40.54	-55.05	-24.40	27.03	-36.72	-13.28	1.35	-2.14	-0.80	1.35	-2.63	-1.29
27	1.82	-2.05	-0.23	0.00	0.00	0.00	0.00	0.00	0.00	0.00	0.00	0.00	0.00	0.00	0.00
28	13.64	-4.82	10.20	0.00	0.00	0.00	0.00	0.00	0.00	0.00	0.00	0.00	0.00	0.00	0.00
29	25.00	-15.91	12.12	16.59	-11.31	6.34	0.00	0.00	0.00	0.00	0.00	0.00	0.00	0.00	0.00
30	7.39	-3.72	3.97	5.22	-2.68	2.68	0.00	0.32	0.32	0.00	0.32	0.32	0.00	0.32	0.32
31	16.52	-11.26	6.30	10.09	-9.13	1.07	1.30	-2.85	-1.57	1.30	-2.85	-1.57	1.30	-2.85	-1.57
32	18.70	-13.76	6.07	12.17	-11.30	0.99	3.48	-4.25	-0.80	0.43	-0.44	0.00	0.00	0.00	0.00
33	0.00	0.00	0.00	0.00	0.00	0.00	0.00	0.00	0.00	0.00	0.00	0.00	0.00	0.00	0.00
34	0.00	0.00	0.00	0.00	0.00	0.00	0.00	0.00	0.00	0.00	0.00	0.00	0.00	0.00	0.00
35	7.81	-9.06	-1.35	5.00	-4.92	0.08	4.69	-5.78	-1.15	0.63	-1.15	-0.53	0.00	0.00	0.00
*Avg*_*s*_	11.28	-10.74	0.71	9.08	-9.47	-0.34	5.76	-6.24	-0.29	0.76	-1.24	-0.50	0.26	-0.68	-0.43

**Table 5:** Computational results for medium-size instances with different values of λ.

	0.5% quantile			1% quantile			2% quantile			5% quantile			10% quantile		
	%*s*	*Gap*_*U*_	*Save*_*U*_	%*s*	*Gap*_*U*_	*Save*_*U*_	%*s*	*Gap*_*U*_	*Save*_*U*_	%*s*	*Gap*_*U*_	*Save*_*U*_	%*s*	*Gap*_*U*_	*Save*_*U*_
36	0.00	0.00	0.00	0.00	0.00	0.00	0.00	0.00	0.00	0.00	0.00	0.00	0.00	0.00	0.00
37	20.97	-2.02	23.98	8.71	-0.32	9.19	0.00	0.00	0.00	0.00	0.00	0.00	0.00	0.00	0.00
38	54.84	-12.36	94.05	23.87	-6.25	23.14	2.58	-2.15	0.45	3.23	-2.71	0.54	0.00	0.00	0.00
39	0.00	0.00	0.00	0.00	0.00	0.00	0.00	0.00	0.00	0.00	0.00	0.00	0.00	0.00	0.00
40	4.05	-5.40	-1.40	0.00	0.04	0.04	0.00	0.02	0.02	0.00	0.03	0.03	0.00	0.03	0.03
41	29.73	-39.17	-13.44	17.57	-24.14	-7.97	1.35	0.28	1.66	0.00	0.00	0.00	0.00	0.00	0.00
42	4.32	-1.62	2.82	3.60	-1.00	2.69	0.00	1.20	1.20	0.00	1.40	1.40	0.00	1.20	1.20
43	10.79	-2.24	9.58	2.88	-0.44	2.51	0.00	0.39	0.39	0.00	0.18	0.18	0.00	0.18	0.18
44	30.94	-19.69	16.28	15.11	-14.56	0.65	2.16	-12.33	-10.40	1.44	-10.73	-9.43	1.44	-7.53	-6.18
*Avg*_*m*_	17.29	-9.17	14.65	7.97	-5.19	3.36	0.68	-1.40	-0.74	0.52	-1.31	-0.81	0.16	-0.68	-0.53

**Table 6:** Computational results for large-size instances with different values of λ.

	0.5% quantile			1% quantile			2% quantile			5% quantile			10% quantile		
	%*s*	*Gap*_*U*_	*Save*_*U*_	%*s*	*Gap*_*U*_	*Save*_*U*_	%*s*	*Gap*_*U*_	*Save*_*U*_	%*s*	*Gap*_*U*_	*Save*_*U*_	%*s*	*Gap*_*U*_	*Save*_*U*_
45	1.02	-1.13	-0.12	0.51	0.11	0.62	0.00	2.74	2.74	0.00	2.25	2.25	0.00	2.41	2.41
46	6.09	-9.69	-3.83	0.51	-0.26	0.25	0.00	3.00	3.00	0.00	3.25	3.25	0.00	3.32	3.32
47	38.07	-62.57	-39.57	16.55	-32.36	-18.95	0.51	1.69	2.21	0.00	1.62	1.62	0.00	1.55	1.55
48	0.00	3.81	3.81	0.00	3.84	3.84	0.00	3.78	3.78	0.00	3.78	3.78	0.00	3.75	3.75
49	0.00	4.74	4.74	0.00	5.36	5.36	0.00	5.97	5.97	0.00	4.95	4.95	0.00	4.74	4.74
50	6.25	2.76	9.61	0.16	7.02	7.19	0.08	6.88	6.97	0.00	6.82	6.82	0.00	6.90	6.90
51	0.00	2.56	2.56	0.00	2.81	2.81	0.00	2.67	2.67	0.00	2.15	2.15	0.00	2.43	2.43
52	0.00	4.75	4.75	0.00	4.81	4.81	0.00	5.10	5.10	0.00	4.08	4.08	0.00	4.44	4.44
53	8.23	-2.82	5.89	0.87	1.31	2.19	0.69	2.31	3.03	0.00	6.56	6.56	0.00	6.41	6.41
54	0.00	7.81	7.81	0.00	7.72	7.72	0.00	7.92	7.92	0.00	8.30	8.30	0.00	8.68	8.68
55	0.00	8.45	8.45	0.00	8.61	8.61	0.00	8.48	8.48	0.00	8.50	8.50	0.00	8.31	8.31
56	8.33	-0.76	8.26	2.59	1.59	4.29	0.29	2.34	2.63	0.00	5.72	5.72	0.00	5.78	5.78
57	11.64	-11.44	0.22	1.82	-4.97	-3.20	1.57	-3.10	-1.55	1.26	-2.03	-0.78	0.00	1.40	1.40
58	33.96	-54.90	-31.70	2.33	-7.27	-5.07	2.20	-6.99	-4.89	2.20	-6.96	-4.87	0.00	0.90	0.90
59	49.37	-79.15	-58.82	38.81	-68.02	-47.75	18.24	-33.63	-18.82	8.36	-16.42	-8.79	4.72	-5.28	-0.59
60	0.98	-3.11	-2.15	0.39	0.18	0.57	0.00	5.71	5.71	0.00	5.85	5.85	0.00	5.98	5.98
61	5.40	-3.67	1.83	0.52	-1.11	-0.59	0.00	2.88	2.88	0.00	2.76	2.76	0.00	2.71	2.71
62	9.17	-17.98	-9.70	0.29	2.68	2.98	0.13	3.25	3.39	0.00	3.86	3.86	0.00	3.96	3.96
63	3.85	-7.12	-3.40	0.98	-1.11	-0.14	0.70	2.82	3.55	0.84	3.59	4.47	0.00	4.19	4.19
64	12.24	-9.42	3.21	6.64	-1.44	5.57	4.55	1.15	5.97	0.63	-0.62	0.01	0.00	1.79	1.79
65	21.68	-35.24	-17.32	18.95	-35.48	-20.40	12.94	-23.76	-12.43	9.09	-12.48	-3.73	1.05	-1.22	-0.17
*Avg*_*l*_	10.30	-12.58	-5.02	4.38	-5.05	-1.87	1.99	0.06	1.82	1.07	1.69	2.70	0.27	3.48	3.76

**Fig 3:**
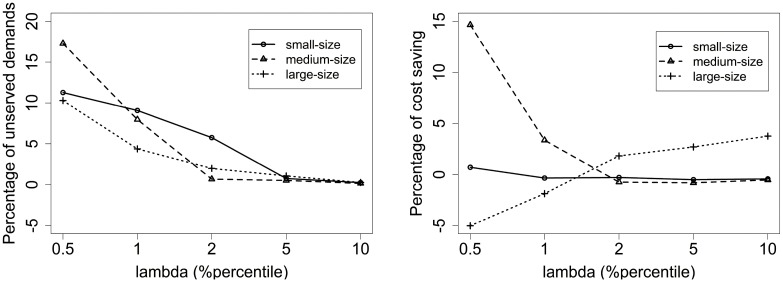
(a) The average percentage of unserved demands for small-size, medium-size and large-size instances; (b) The average percentage of cost saving for small-size, medium-size and large-size instances.

From Tables 4, 5 and 6, we can conclude that the number of unserved demands becomes smaller as λ grows, while the length of routes becomes larger. Fig 3(a) shows the average percentage of unserved demands for small-size, medium-size, and large-size instances. It can be seen that the unserved demands are close to zero with λ increasing. Fig 3(b) shows the average percentage of cost-saving. For small-size instances, small values of λ lead to lots of unserved demands and poor values of cost-saving. The best cost-saving achieves at λ = 5% quantile. Similar conclusions can be found for medium-size instances. For large-size instances, it is better to set λ small to shorten routes length by scarifying some demands. The best value is achieved at λ = 0.5% quantile. That is what we use in Section 5.1.

## 6 A case study: Hangzhou bike-sharing system

In this section, we provide a case study, Hangzhou bike-sharing system, to verify our proposed approach further. The dataset is provided by Hangzhou Public Transport Ltd. and is available as the supplementary material in S3 Appendix. We forecast customer demands by the random forest and solve best-found routes by the distributed ACO algorithm in Section 4. Moreover, we adopt actual distances instead of Euclidean distances. Thus, our method is of practical interests in real-world applications.

### 6.1 Data description

Hangzhou, one of the earliest cities to develop BSS in China, started a test operation on May 1, 2008, and officially operated on September 16, 2008. At the end of 2018, it has 3,354 bike stations and 86,800 bikes. The maximum rental volume reaches 473,300 bikes each day, and the total rental volume exceeds 737 million bikes. We select a subset of transaction data between August 9, 2013, and November 12, 2013, in Xiasha District, Hangzhou.

**Fig 4:**
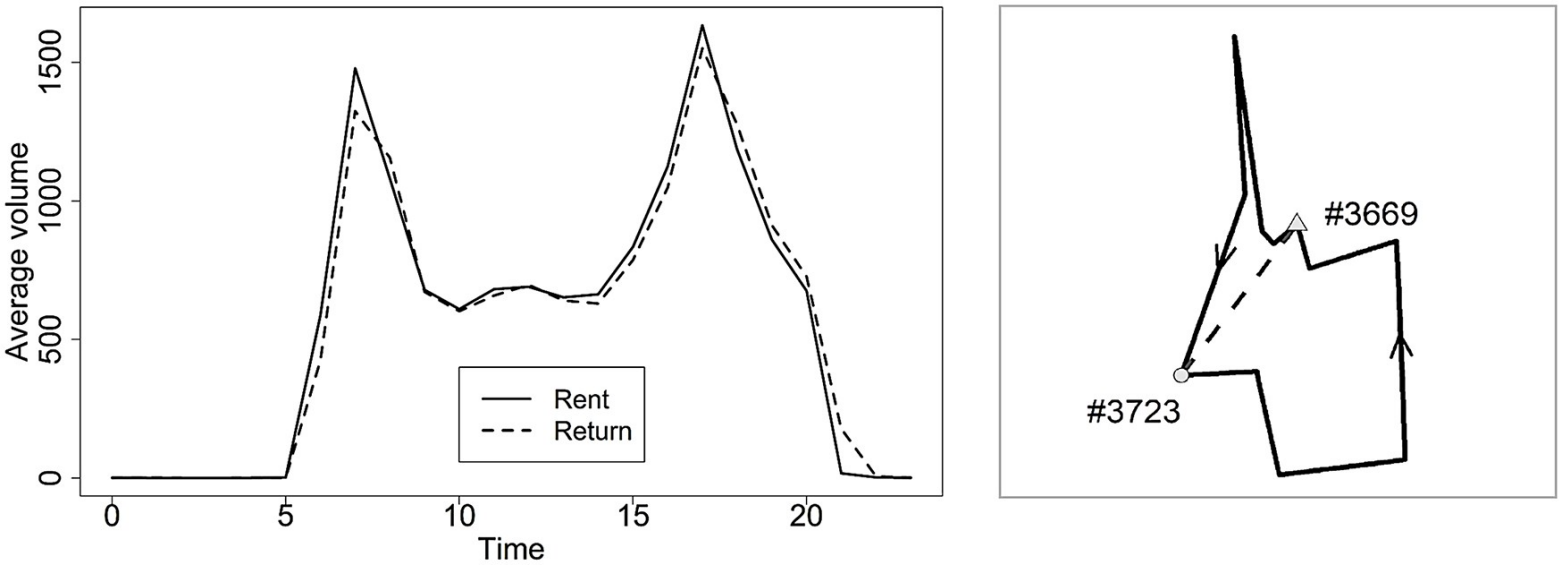
(a) Average volumes of the rent (return) per hour in Xiasha from August 9, 2013 to November 12, 2013; (b) The actual shortest driving paths in the opposite directions (solid) and the straight path (dashed) between the station #3669 and the station #3723.

There are 151 bike stations in Xiasha. Fig 4(a) is a line chart of the average hourly volume of the rent (return). Users mainly use bikes from 6:00 to 22:00. The volume of the rent (return) per hour during 8:00-20:00 is in the range of [600, 1000], and the peak appears at two periods, that is, 7:00-8:00 and 17:00-18:00, when the amount reaches around 1400. Taking the peak in the morning (7:00-8:00) as an example, we use the method in Section 4 to forecast the demands during this period in the next morning and determine the operational scheme.

We also need obtain the distance matrix *D* = (*d*_*ij*_)_(*n*+1)×(*n*+1)_. Note that the vehicles need to serve stations along the road, and the distance between the stations cannot be replaced by the Euclidean distance. Moreover, the distance matrix is asymmetric because of the one-way direction principle. The Gaode map (https://lbs.amap.com/api) provides an API interface for obtaining the driving distance between any two locations. Here we use Python to call the Gaode map web service API to get the shortest driving distance between stations. Taking the station #3669 and the station #3723 as examples, Fig 4(b) shows the differences between the shortest driving distance in the opposite directions and the simple Euclidean distance. It can be seen that they are quite different. Acquiring the shortest driving distance between 151 stations, we find the average is 6.04 kilometers, while the average Euclidean distance is only 3.25 kilometers. The shortest driving distance is much larger than the Euclidean distance.

### 6.2 Forecasting customer demands

Denote $y_{i}^{\left(t\right)}$ as the demands during the peak period in the morning and $x_{i}^{\left(t\right)}$ as the *p*-dimensional vector of predictors of the station *i* on the *t*-th day. Calculating the autocorrelation coefficients of the sequence $\{y_{i}^{\left(t\right)},t=1,\cdots ,96\},i=1,\cdots ,151$, we find that the autocorrelation can be traced back to the 8-th order, thus the historical demands of the first 8 days of the station *i* are included in the predictors of the *t*-th day. Due to the time lag, the new dataset covers 88 days starting from the ninth day, August 17 to November 12. In addition to the demands of the first 8 days, we crawled the weather records of the corresponding period from the weather website (lishi.tianqi.com.), including the temperature (°C), the dew point (°C), the humidity (%), the air pressure (hundreds of Pascals), the wind speed (km/h) and whether it rained (binary variable). Moreover, we also add the day of the week and whether it is a holiday (binary variable) to the predictors as the date information. Table 7 gives a description of the predictors.

**Table 7:** Descriptions of predictors.

Predictor	Description	Predictor	Description
*x*_*i*1_	the historical demand 1 day ago	*x*_*i*12_	whether it is Thursday
*x*_*i*2_	the historical demand 2 days ago	*x*_*i*13_	whether it is Friday
*x*_*i*3_	the historical demand 3 days ago	*x*_*i*14_	whether it is Saturday
*x*_*i*4_	the historical demand 4 days ago	*x*_*i*15_	whether it is holiday
*x*_*i*5_	the historical demand 5 days ago	*x*_*i*16_	the temperature (°C)
*x*_*i*6_	the historical demand 6 days ago	*x*_*i*17_	the dew point (°C)
*x*_*i*7_	the historical demand 7 days ago	*x*_*i*18_	the humidity (%)
*x*_*i*8_	the historical demand 8 days ago	*x*_*i*19_	the air pressure (hundreds of Pascals)
*x*_*i*9_	whether it is Monday	*x*_*i*20_	the wind speed (km/h)
*x*_*i*10_	whether it is Tuesday	*x*_21_	whether it rained
*x*_*i*11_	whether it is Wednesday		

**Fig 5:**
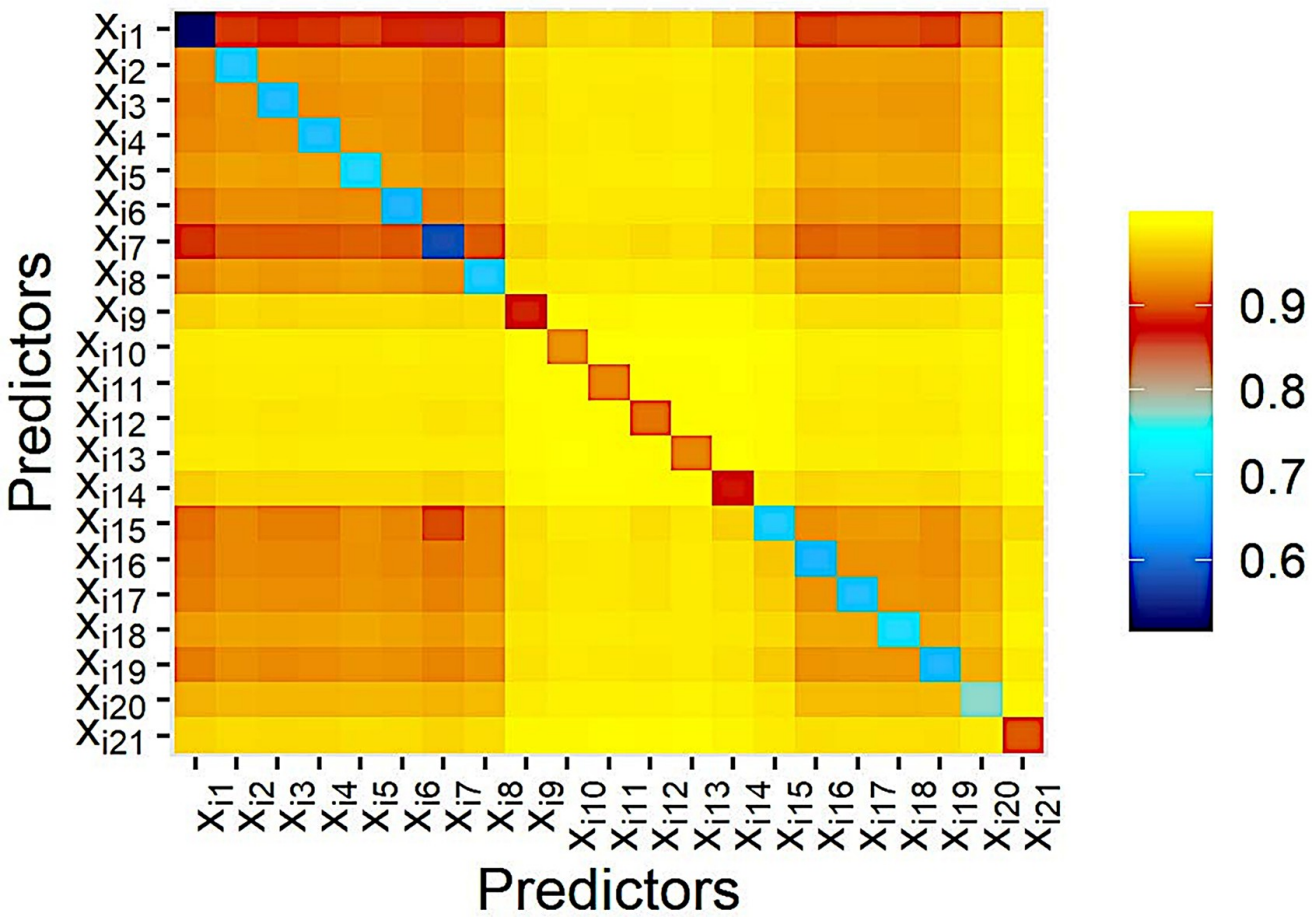
Average results of the maximal subtree analysis over 151 stations. Small entries on the diagonal indicate predictive variables and a small entry on the triangle is a sign of an interaction between the two variables.

To explore the interactive effects between factors, we invoke a maximal subtree analysis proposed by [52]. The result of the analysis is a matrix where entries on the diagonal *μ*_*jj*_ represent the minimal normalized depth of the *j*-th variable relative to the root node and entries on the triangle $\mu_{j_{1}j_{2}}$ indicate the minimal normalized depth of the *j*_2_-th variable relative to the maximal subtree for the *j*_1_-th variable. Small entries on the diagonal indicate predictive variables and a small entry on the triangle is a sign of an interaction between the two variables. Fig 5 shows the average results over the 151 stations. One can see that entries on the triangle are close to 1. Thus there is no significant interaction between the variables. Besides, we find the historical data, whether it is holiday, the temperature, the dew point, the humidity, the air pressure, and the wind speed are important explanatory variables to forecast demands.

**Fig 6:**
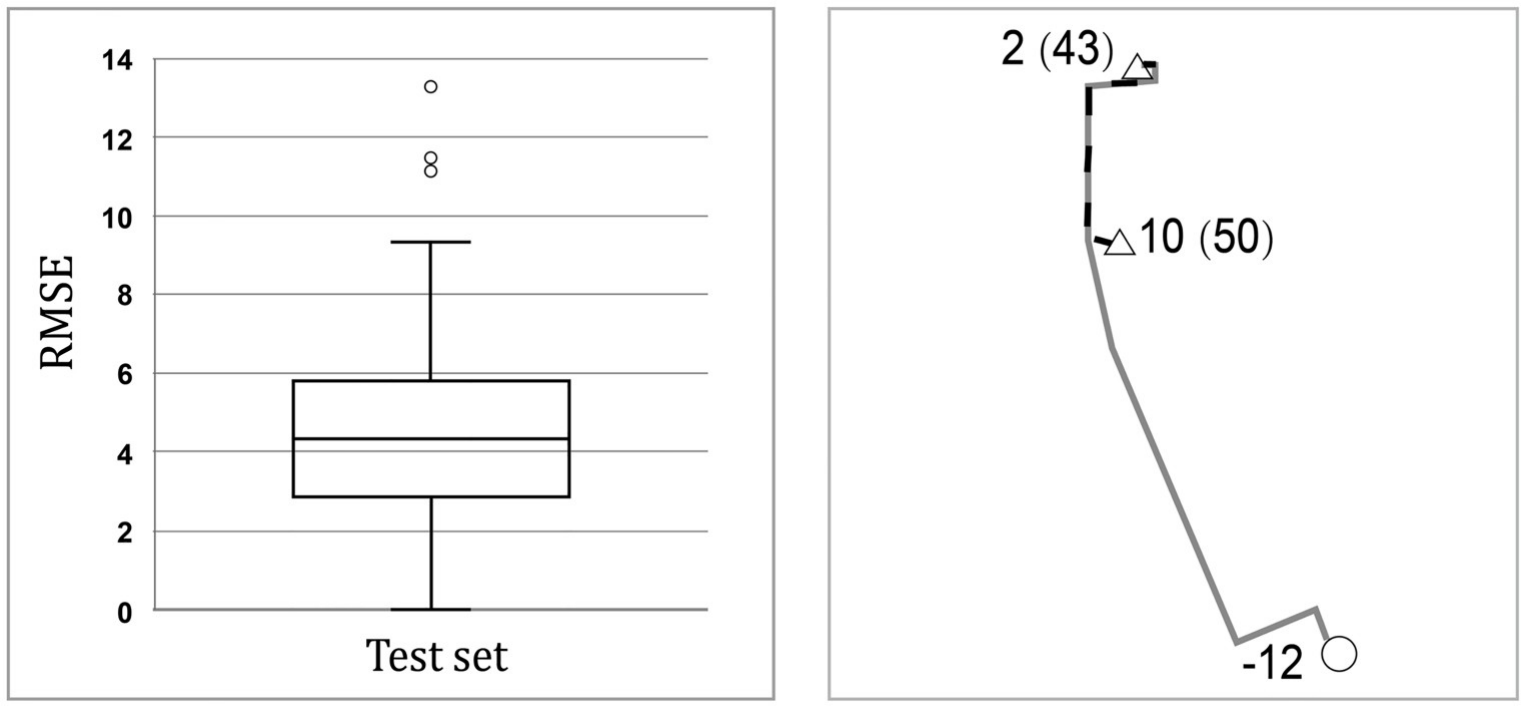
(a) The RMSE of demands forecasting on the test set; (b) A part of the best found route (solid line) and the path (dashed line) to the nearest feasible station having bikes to be delivered.

We use the data of the first 67 days, from August 17 to October 22, ${\left\{{\left(x_{i}^{\left(t\right)}\right)}^{T},y_{i}^{\left(t\right)}\right\}}_{t=1}^{67}$ as a training set to fit the random forest model described in Algorithm 1, and the data of the last 21 days, from October 23 to November 12, as a test set to calculate the forecasting error. The boxplot of the RMSE of all stations is shown in Fig 6(a). We see that the RMSE is concentrated in the interval [0, 6], and only three stations have an RMSE of more than 10. The average RMSE of all stations is 4.41, and the median is 4.37. The results show that the random forest model has an excellent performance in forecasting demands.

Finally, we apply the fitted random forest model to predict the demands during the peak period in the morning on November 13, 2013. During the peak period, the total demand (∑|*y*_*i*_|) is 1033. Five hundred fifty-five bikes need to be picked up from 68 stations. The number of bikes to be picked up falls in the interval [1, 26], with an average of 8.16 and a median of 8. Moreover, 478 bikes need to be delivered to 71 stations. The number of bikes to be delivered occurs in the interval [-22,-1] by an average -6.73 and a median -5. Moreover, demands forecasts of 12 stations are 0, and these stations will be removed before the route construction.

### 6.3 Finding the routing scheme

After obtaining the actual driving distance between stations and the customer demands of each station, we use the proposed algorithm to solve the optimization problem. Removing the 12 stations where the demands are predicted as 0, the number of stations that need to be served is 139.

The first category of parameters related to the algorithm itself is the same as those in Section 5.1. According to the information we obtain from the BSS company, the number of vehicles is set as *K* = 3, and the capacity of vehicles is *C* = 50. The depot is located at the same place as the station #3707, which is near the center of Xiasha district. We apply the proposed ACO algorithm to solve the BRP (10)–(18) with different values of λ. The results are shown in Table 8. It can be seen that the length of routes is increasing as λ grows, while the number of unserved demands is decreasing. When λ = 10%, the unserved demand is zero, and the best-found route length, denoted as *L*_*ub*_, is 285.06 km. For other values of λ, we give the results of percentage of unserved demands (%*s*), save of route length (*Gap*_*U*_) and save of cost per bike (*Save*_*U*_) compare with *L*_*ub*_, where there is no unserved demand.

**Table 8:** Results for BSS in Xiasha, Hangzhou.

0.5% quantile			1% quantile			2% quantile			5% quantile			10% quantile	
%*s*	*Gap*_*U*_	*Save*_*U*_	%*s*	*Gap*_*U*_	*Save*_*U*_	%*s*	*Gap*_*U*_	*Save*_*U*_	%*s*	*Gap*_*U*_	*Save*_*U*_	%*s*	*L*_*ub*_
1.65	-4.58	-2.99	0.90	-3.24	-2.36	0.53	-2.26	-1.73	0.32	-2.01	-1.70	0.00	285.06

Since this is a large-size instance, we choose λ as the 0.5% quantile of $\left\{d_{v_{0},i},i\in V\right\}$, where the cost-saving achieves best. The length of routes is reduced by 4.58% with 1.65% unserved demands, compared with the routing scheme without unserved demands. The percentage of cost-saving achieves 2.99%. The proposed method can greatly reduce the length of routes by failing to meet a small part of customer demands.

**Fig 7:**
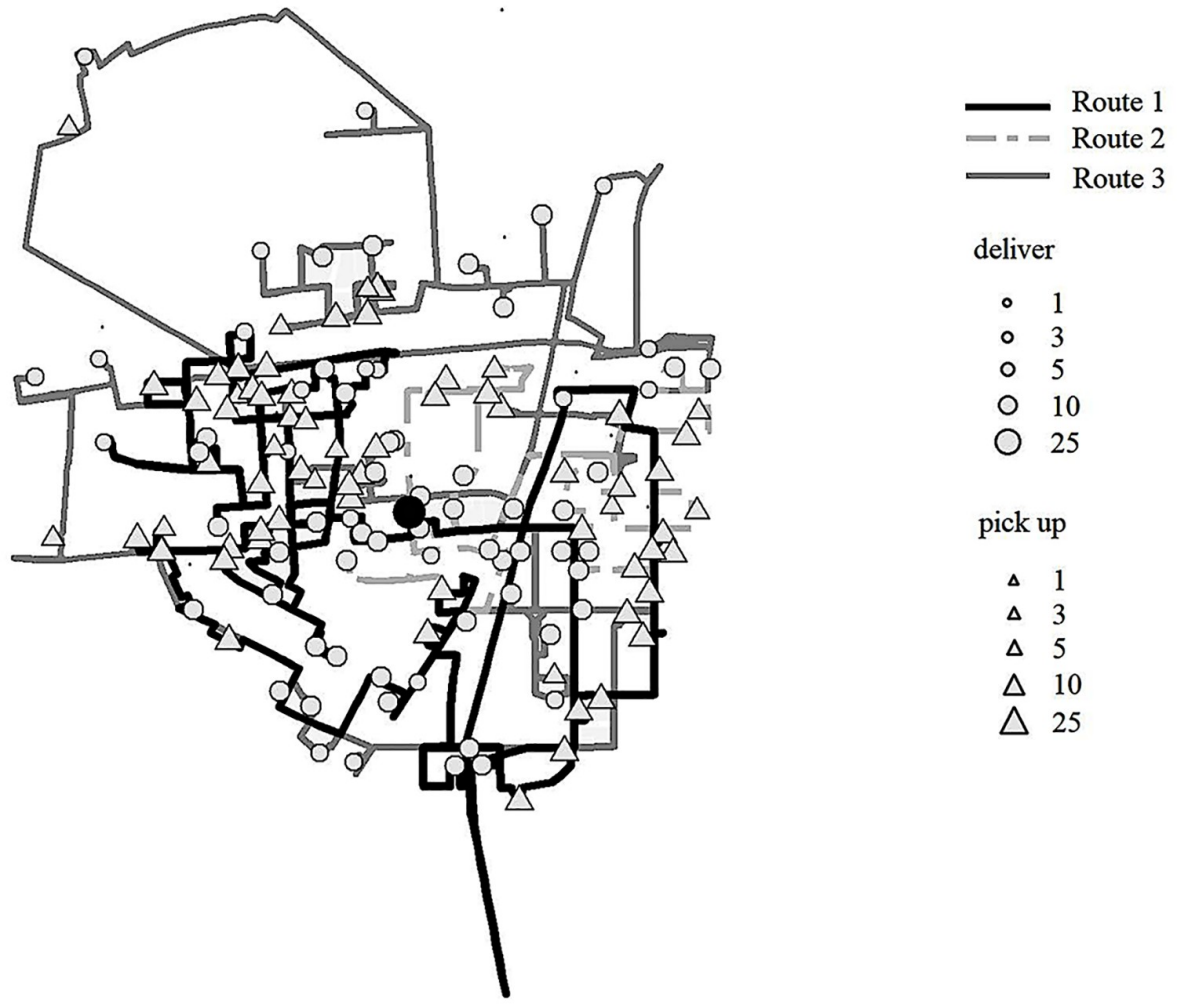
The best-found routes of three vehicles. The black circle marks the depot. The circle in gray represents the station needs bikes to be picked up. The triangle in gray indicates the station needs bikes to be delivered. The best-found routes for three vehicles are shown in different grayscales and line types.

To illustrate the effect of unserved demands, Fig 6(b) shows a part of the best-found route in the dashed line where the circle represents the station needs bikes to be delivered, and the triangle represents the station needs bikes to be picked up. “2 (43)” means the station requires two bikes to be picked up and after the vehicle serving this station, there are 43 bikes carried on the vehicle in the best found scheme. Similarly, “10 (50)” means the station requires ten bikes to be picked up, and after the vehicle serving this station, there are 50 bikes carried on the vehicle. The vehicle serves the station “2 (43)” first and then serve the station “10 (50)”, thus three bikes are unserved in the latter station because of the maximum capacity constraint. If the demands are required to satisfy strictly, the vehicle needs to go to a station having bikes to be delivered first and then go back to serve the station labeled as “10 (50)”. The solid line shows the path from the station labeled as “2 (43)” to the nearest available station having bikes to be delivered, which is labeled as “-12”, indicating the station requires 12 bikes to be delivered. It can be seen that allowing some demands unserved can make the operational scheme more flexible and shorten the length of routes. Fig 7 shows the best-found path for three vehicles in different grayscales and line types. The three vehicles start from the depot represented by the black circle and finally go back to it. The best found initial number of bikes is 3, 45, 1 given by Proposition 2, where each vehicle serves 55, 30, and 54 stations, respectively.

## 7 Conclusions

In this paper, we investigated the bike-sharing rebalancing problem in BSS. Firstly, the random forest algorithm to forecast demands for each station is presented. Secondly, for the route construction task, a new model with unserved demands is proposed. It strikes a trade-off between the length of routes and customer demands fulfillment. A distributed ACO for solving the proposed model is carefully designed, together with several route improvement strategies. Computational results on benchmark instances show the advantage of the new method. Our model and algorithms are of practical interest in real-world applications. To this end, we provided a case study of BSS in Xiasha, Hangzhou.

There are several possible extensions. Firstly, vehicles can be heterogeneous in our model, and the depots can be multiple, which are easily extended in the future. Secondly, one may consider a strategy that allows vehicles to discover and skip the stations with few demands. This can lead to even much shorter routes. Also, we can consider the case where the number of stations to be served is uncertain and varies in different operational schemes. Furthermore, we can separate unserved pickup and delivery demands and thus develop a different variant of the current model.

## Supporting information

S1 Appendix Proof of Proposition 1. (PDF)

S2 Appendix Proof of Proposition 2. (PDF)

S3 Appendix Hangzhou dataset. (ZIP)
