# Supplementary material for: Distributed forecasting and ant colony optimization for the bike-sharing rebalancing problem with unserved demands
Source: PLoS One. 2019 Dec 31;14(12):e0226204. doi: 10.1371/journal.pone.0226204 (PMC6938368; doi:10.1371/journal.pone.0226204)
Supplement: S1 Appendix — (PDF) [file pone.0226204.s001.pdf]

Supporting information for "Distributed forecasting and ant colony optimization for the bike-sharing rebalancing problem with unserved demands"

Yiwei Fan[1,2], Gang Wang[3], Xiaoling Lu[1,2*], Gaobin Wang[4]

1 Center for Applied Statistics, Renmin University of China, Beijing, China
2 School of Statistics, Renmin University of China, Beijing, China
3 Department of Decision & Information Sciences, Charlton College of Business, University of Massachusetts Dartmouth, MA, USA
4 Invesco Great Wall Fund Management, Shenzhen, China

* Corresponding author: xiaolinglu@ruc.edu.cn

S1 Appendix.

Proof of Proposition 1. For the BRP without unserved demands, considering the route $o = \{o_1, o_2, \cdots, o_{|o|}\}$, if the route is feasible, then we have $f_{o_1 o_2} = z, f_{o_i o_{i+1}} = z + \sum_{j=2}^{i} y_{o_j}, i = 2, \cdots, |o| - 1$. Considering the maximum capacity constraint, we find that

$$0 \leq z \leq C \tag{S.1}$$

$$0 \leq z + \sum_{j=2}^{i} y_{o_j} \leq C, i = 2, \cdots, |o| - 1. \tag{S.2}$$

Rewrite the inequality (S.2) as $-\sum_{j=2}^{i} y_{o_j} \leq z \leq C - \sum_{j=2}^{i} y_{o_j}, i = 2, \cdots, |o| - 1$. Note that it holds for any $2 \leq i \leq |o| - 1$. Thus, we have $\max_{2 \leq i \leq |o|-1}\{-\sum_{j=2}^{i} y_{o_j}\} \leq z \leq \min_{2 \leq i \leq |o|-1}\{C - \sum_{j=2}^{i} y_{o_j}\}$. That is, $-\min_{2 \leq i \leq |o|-1}\{\sum_{j=2}^{i} y_{o_j}\} \leq z \leq C - \max_{2 \leq i \leq |o|-1}\{\sum_{j=2}^{i} y_{o_j}\}$. Combining with (S.1), we have $-\min\{0, \min_{2 \leq i \leq |o|-1} \sum_{j=2}^{i} y_{o_j}\} \leq z \leq C - \max\{0, \max_{2 \leq i \leq |o|-1} \sum_{j=2}^{i} y_{o_j}\}$.

The route is feasible if and only if there is a feasible solution for z, that is, $-\min\{0, \min_{2 \leq i \leq |o|-1} \sum_{j=2}^{i} y_{o_j}\} \leq C - \max\{0, \max_{2 \leq i \leq |o|-1} \sum_{j=2}^{i} y_{o_j}\}$. Thus, we have $C - \max\{0, \max_{2 \leq i \leq |o|-1} \sum_{j=2}^{i} y_{o_j}\} + \min\{0, \min_{2 \leq i \leq |o|-1} \sum_{j=2}^{i} y_{o_j}\} \geq 0$.

This completes the proof. □
