# Supplementary material for: Distributed forecasting and ant colony optimization for the bike-sharing rebalancing problem with unserved demands
Source: PLoS One. 2019 Dec 31;14(12):e0226204. doi: 10.1371/journal.pone.0226204 (PMC6938368; doi:10.1371/journal.pone.0226204)
Supplement: S2 Appendix — (PDF) [file pone.0226204.s002.pdf]

Supporting information for "Distributed forecasting and ant colony optimization for the bike-sharing rebalancing problem with unserved demands"

Yiwei Fan[1,2], Gang Wang[3], Xiaoling Lu[1,2*], Gaobin Wang[4]

1 Center for Applied Statistics, Renmin University of China, Beijing, China
2 School of Statistics, Renmin University of China, Beijing, China
3 Department of Decision & Information Sciences, Charlton College of Business, University of Massachusetts Dartmouth, MA, USA
4 Invesco Great Wall Fund Management, Shenzhen, China

* Corresponding author: xiaolinglu@ruc.edu.cn

S2 Appendix.

Proof of Proposition 2. Considering the route $o = \{o_1, o_2, \cdots, o_{|o|}\}$, we first prove that when z increasing, the flow $f_{o_i o_{i+1}}$ is non-decreasing for any $2 \le i \le |o| - 1$ by induction. For any $2 \le i \le |o| - 1$, one can verify that if $y_{o_i} \le 0$,

$$f_{o_i o_{i+1}} = \begin{cases} f_{o_{i-1} o_i} + y_{o_i}, & \text{if } f_{o_{i-1} o_i} > -y_{o_i} \\ 0, & \text{if } f_{o_{i-1} o_i} \le -y_{o_i}. \end{cases} \tag{S.1}$$

If $y_{o_i} > 0$,

$$f_{o_i o_{i+1}} = \begin{cases} f_{o_{i-1} o_i} + y_{o_i}, & \text{if } f_{o_{i-1} o_i} \le C - y_{o_i} \\ C, & \text{if } f_{o_{i-1} o_i} > C - y_{o_i}. \end{cases} \tag{S.2}$$

Then $f_{o_i o_{i+1}}$ is non-decreasing when $f_{o_{i-1} o_i}$ increasing. Combing with the fact that $f_{o_1 o_2} = z$, we conclude that $f_{o_i o_{i+1}}$ is non-decreasing when z increasing for any $2 \le i \le |o| - 1$. Note that $f_{o_i o_{i+1}} - f_{o_{i-1} o_i} = y_{o_i} - s_{o_i}$, it holds that if $y_{o_i} \le 0$,

$$s_{o_i} = \begin{cases} 0, & \text{if } f_{o_{i-1} o_i} > -y_{o_i} \\ y_{o_i} + f_{o_{i-1} o_i}, & \text{if } f_{o_{i-1} o_i} \le -y_{o_i}. \end{cases} \tag{S.3}$$

If $y_{o_i} > 0$,

$$s_{o_i} = \begin{cases} 0, & \text{if } f_{o_{i-1} o_i} \le C - y_{o_i} \\ y_{o_i} - C + f_{o_{i-1} o_i}, & \text{if } f_{o_{i-1} o_i} > C - y_{o_i}. \end{cases} \tag{S.4}$$

Thus, we have s_{o_i} is non-decreasing when z increasing.

The following equations can be derived from (S.1)-(S.4),

$$s_{o_2} = \begin{cases} \min\{0, z + y_{o_i}\}, \text{ if } y_{o_i} \le 0 \\ \max\{0, z + y_{o_i} - C\}, \text{ if } y_{o_i} > 0 \end{cases} \tag{S.5}$$

$$s_{o_i} = \begin{cases} \min\{0, z + \sum_{j=2}^{i} y_{o_j} - \sum_{j=2}^{i-1} s_{o_j}\}, \text{ if } y_{o_i} \le 0 \\ \max\{0, z + \sum_{j=2}^{i} y_{o_j} - \sum_{j=2}^{i-1} s_{o_j} - C\}, \text{ if } y_{o_i} > 0 \end{cases}, i = 3, \cdots, |o| - 1, \tag{S.6}$$

$$f_{o_i o_{i+1}} = z + \sum_{j=2}^{i} y_{o_j} - \sum_{j=2}^{i} s_{o_j}, i = 2, \cdots, |o| - 1. \tag{S.7}$$

We consider the case $\arg\max\limits_{2\le i\le i_1}\sum\limits_{j=2}^{i} y_{o_j} < \arg\min\limits_{2\le i\le i_1}\sum\limits_{j=2}^{i} y_{o_j}$. The proof for the opposite case is similar and is omitted for simplicity. By the definition of i_1, we know that $i_1 = \arg\min\limits_{2\le i\le i_1}\sum\limits_{j=2}^{i} y_{o_j}$. Denote $i_2 = \arg\max\limits_{2\le i\le i_1}\sum\limits_{j=2}^{i} y_{o_j}$. We prove that the total value of unserved demands $\sum_{i=2}^{|o|-1}|s_{o_i}|$ is non-increasing when $z \le C - \max\{0, \max\limits_{2\le i\le i_1}\sum\limits_{j=2}^{i} y_{o_j}\}$ increasing and non-decreasing when $z \ge C - \max\{0, \max\limits_{2\le i\le i_1}\sum\limits_{j=2}^{i} y_{o_j}\}$ increasing.

We first prove the total unserved demands is non-decreasing with $z \ge C - \max\{0, \max\limits_{2\le i\le i_1}\sum\limits_{j=2}^{i} y_{o_j}\}$ increasing. By the definition of i_2, it can be seen $y_{o_{i_2}} \ge 0$. Note that the route is feasible for $o_2, \cdots, o_{i_2}$ by the definition of i_1 and the fact that $i_2 < i_1$. From Proposition 1, for z in $[-\min\{0, \min\limits_{2\le i\le i_2}\sum\limits_{j=2}^{i} y_{o_j}\}, C - \max\{0, \sum\limits_{j=2}^{i_2} y_{o_j}\}]$, it holds that $s_{o_i} = 0$ for any $2 \le i \le i_2$. For $y_{o_i} \le 0, 2 \le i \le i_2$, because $s_{o_i} \le 0$ is non-decreasing with z increasing, we have when $z \ge C - \max\{0, \max\limits_{2\le i\le i_1}\sum\limits_{j=2}^{i} y_{o_j}\} = C - \max\{0, \sum\limits_{j=2}^{i_2} y_{o_j}\}$, $s_{o_i} = 0$. Thus, by (S.6)

$$\begin{aligned}\sum_{y_{o_j}>0}^{i_2} s_{o_j} &= \max\left\{\sum_{y_{o_j}>0}^{i_2-1} s_{o_j}, z + \sum_{j=2}^{i_2} y_{o_j} - C\right\}\\ &= \max\left\{0, \max_{2\le i\le i_2}\left\{z + \sum_{j=2}^{i} y_{o_j} - C\right\}\right\} = z + \sum_{j=2}^{i_2} y_{o_j} - C.\end{aligned}$$

Based on (S.7), we have $f_{o_{i_2}o_{i_2+1}} = C$. Then we know that unserved demands for $o_i, i > i_2$ are unchanged for any z. And the total value of unserved demands $\sum_{j=2}^{|o|-1}|s_{o_j}| = |\sum_{y_{o_j}>0}^{i_2} s_{o_j}| + \sum_{j=i_2+1}^{|o|-1}|s_{o_j}|$ is non-decreasing with $z \ge C - \max\{0, \max\limits_{2\le i\le i_1}\sum\limits_{j=2}^{i} y_{o_j}\}$ increasing.

We then prove the total unserved demands is non-increasing with $z \le C - \max\{0, \max\limits_{2\le i\le i_1}\sum\limits_{j=2}^{i} y_{o_j}\}$ increasing. By the definition of i_1, it can be seen $y_{o_{i_1}} \le 0$. Note that the route is feasible for $o_2, \cdots, o_{i_1-1}$ by the definition of i_1. From Proposition 1, for z in $[-\min\{0, \min\limits_{2\le i\le i_1-1}\sum\limits_{j=2}^{i} y_{o_j}\}, C - \max\{0, \sum\limits_{j=2}^{i_2} y_{o_j}\}]$, it holds that $s_{o_i} = 0$ for any $2 \le i \le i_1 - 1$. For $y_{o_i} > 0, 2 \le i \le i_1 - 1$, because $s_{o_i} \ge 0$ is non-decreasing with z increasing, we have when $z \le C - \max\{0, \max\limits_{2\le i\le i_1}\sum\limits_{j=2}^{i} y_{o_j}\} < -\min\{0, \min_{2\le i\le i_1}\sum_{j=2}^{i} y_{o_j}\}$, $s_{o_i} = 0$ for any $y_{o_i} > 0, 2 \le i \le i_1$. Thus, by (S.6)

$$\begin{aligned}\sum_{y_{o_j}\le 0}^{i_1} s_{o_j} = \min\left\{\sum_{y_{o_j}\le 0}^{i_1-1} s_{o_j}, z + \sum_{j=2}^{i_1} y_{o_j}\right\} &= \min\left\{0, \min_{2\le i\le i_1}\left\{z + \sum_{j=2}^{i} y_{o_j}\right\}\right\}\\ &= z + \sum_{j=2}^{i_1} y_{o_j}.\end{aligned}$$

Based on (S.7), we have $f_{o_{i_1} o_{i_1+1}} = 0$. Then the unserved demands for $o_i, i > i_1$ are unchanged for any z. And $\sum_{j=2}^{|o|-1} |s_{o_j}| = |\sum_{y_{o_j} \leq 0}^{i_1} s_{o_j}| + \sum_{j=i_1+1}^{|o|-1} |s_{o_j}|$ is non-increasing with $z \leq C - \max\{0, \max\limits_{2 \leq i \leq i_1} \sum\limits_{j=2}^{i} y_{o_j}\}$ increasing.

Thus we have $z = \max\{0, C - \max\{0, \max\limits_{2 \leq i \leq i_1} \sum\limits_{j=2}^{i} y_{o_j}\}\}$.

This completes the proof. □
